# Discrete- vs. Continuous-Time Modeling of Unequally Spaced Experience Sampling Method Data

**DOI:** 10.3389/fpsyg.2017.01849

**Published:** 2017-10-20

**Authors:** Silvia de Haan-Rietdijk, Manuel C. Voelkle, Loes Keijsers, Ellen L. Hamaker

**Affiliations:** ^1^Methodology and Statistics for the Behavioural, Biomedical and Social Sciences, Utrecht University, Utrecht, Netherlands; ^2^Center for Lifespan Psychology, Max Planck Institute for Human Development, Berlin, Germany; ^3^Psychological Methods, Humboldt University Berlin, Berlin, Germany; ^4^Developmental Psychology, Tilburg University, Tilburg, Netherlands; ^5^Quantitative Psychology and Individual Differences, Catholic University Leuven, Leuven, Belgium

**Keywords:** experience sampling method, autoregressive modeling, continuous-time, discrete-time, unequal spacing, intensive longitudinal data, time series analysis

## Abstract

The Experience Sampling Method is a common approach in psychological research for collecting intensive longitudinal data with high ecological validity. One characteristic of ESM data is that it is often unequally spaced, because the measurement intervals within a day are deliberately varied, and measurement continues over several days. This poses a problem for discrete-time (DT) modeling approaches, which are based on the assumption that all measurements are equally spaced. Nevertheless, DT approaches such as (vector) autoregressive modeling are often used to analyze ESM data, for instance in the context of affective dynamics research. There are equivalent continuous-time (CT) models, but they are more difficult to implement. In this paper we take a pragmatic approach and evaluate the practical relevance of the violated model assumption in DT AR(1) and VAR(1) models, for the *N* = 1 case. We use simulated data under an ESM measurement design to investigate the bias in the parameters of interest under four different model implementations, ranging from the true CT model that accounts for all the exact measurement times, to the crudest possible DT model implementation, where even the nighttime is treated as a regular interval. An analysis of empirical affect data illustrates how the differences between DT and CT modeling can play out in practice. We find that the size and the direction of the bias in DT (V)AR models for unequally spaced ESM data depend quite strongly on the true parameter in addition to data characteristics. Our recommendation is to use CT modeling whenever possible, especially now that new software implementations have become available.

## 1. Introduction

Intensive longitudinal research is a popular way of investigating intra-individual processes in psychology, such as the dynamics of emotion, behavior and thought processes from moment to moment. One established approach of investigating such processes is the experience sampling method (ESM; Hektner et al., 2007), in which aspects of people's experiences are measured as they go about their normal lives, using, for instance, smartphone mobile apps. This method results in high ecological validity and can be used to study fluctuations that occur over short time periods, rather than only slow or long term developments. The current study focuses on an unresolved question concerning optimal analysis approaches for the type of intensive data resulting from such research, which is critical to address given the importance of this research methodology.

A search for recent journal articles in which the ESM is employed illustrates the method's popularity and versatility, yielding studies into widely varying topics such as the social experiences of children with Asperger syndrome (Cordier et al., 2016); tourists' experiences (Quinlan Cutler et al., 2016); involuntary musical imagery (Floridou and Müllensiefen, 2015); affect regulation (Catterson et al., 2016); body satisfaction and self-esteem in adolescents (Fuller-Tyszkiewicz et al., 2015); and chronic fatigue symptom fluctuations (Band et al., 2015). The ESM has been most frequently applied, however, in the field of emotion dynamics, mood and psychopathology (e.g., Larson et al., 1980; Ebner-Priemer and Sawitzki, 2007; Brans et al., 2013; for a review of ESM studies in the context of mood disorders, see Ebner-Priemer and Trull, 2009).

By using the ESM, researchers can study lagged relationships between different variables, or the correlation of a variable with itself over time, addressing questions about processes, development or dynamics. An important issue, which to our knowledge has not yet been addressed, is the question whether it is justifiable to use discrete-time (DT) modeling approaches for unequally spaced ESM data. In the broader literature on longitudinal data analysis, it has been pointed out that continuous-time (CT) modeling, which explicitly accounts for the timing of measurements, has several important advantages over DT modeling, both for theoretical and practical reasons (see, e.g., Oud and Delsing, 2010; Voelkle et al., 2012; Deboeck and Preacher, 2016). A specific limitation of DT models, which is particularly relevant for ESM research, is these models' inbuilt assumption that the time intervals between consecutive measurements are all equal. In ESM studies, the intervals are typically varied, with the result that the data clearly violate this assumption. CT modeling does not involve this assumption and can make full use of the information contained not only in the observations themselves, but also in the exact timing of the measurements. Still, DT models continue to be widely used even in these situations.

In the current study, we evaluate the practical relevance of the choice between DT and CT modeling in the specific context of (vector) autoregressive modeling of unequally spaced ESM data. We focus on the *N* = 1 case where a model is fit to one person's data, using simulations as well as an empirical illustration to address the question of whether substantial bias in the parameters of interest may result from using DT (vector) autoregressive models. This is an important question because autoregressive (AR) models and extensions such as the vector autoregressive (VAR) model are frequently used in ESM research in areas such as affect dynamics (e.g., Suls et al., 1998; Koval and Kuppens, 2012; de Haan-Rietdijk et al., 2016; Van Roekel et al., 2016) and the emerging network approach to psychopathology (Borsboom and Cramer, 2013; Bringmann et al., 2013; Wichers, 2014). Thus, questions about the validity of DT model results for unequally spaced data bear directly on findings reported in these fields and on recommendations for follow-up studies, in addition to general questions about optimal design and data analysis for these types of studies. We approach the comparison of different methods from a pragmatic point of view. Our goal is not only to identify the best method for analyzing unequally spaced ESM data, but also to evaluate the robustness of common approaches under conditions that are typical for current practice.

This paper is organized as follows. We start by providing the reader with essential background information through a brief introduction of the ESM as well as the AR / VAR modeling approach, highlighting the problem that can occur when unequally spaced ESM data are analyzed with such models. Then we discuss CT alternatives to these models which would solve this problem, looking also at other considerations regarding the choice between CT and DT modeling for ESM data. After that, we use simulations to investigate the bias that may result when data from a typical ESM design with unequal measurement intervals are analyzed using AR(1) or VAR(1) model implementations. To address the practical significance of this issue, we present an empirical illustration using positive affect ESM data from three adolescents. We conclude with a discussion of the findings and limitations of the current study and recommendations for researchers interested in using the ESM.

## 2. Background: ESM data and (V)AR modeling

In this section we briefly introduce the ESM, and we discuss the discrete-time AR / VAR models which have been used to study lagged relationships in such data, as well as a limitation of these models that is relevant for unequally spaced ESM data.

### 2.1. The experience sampling method

The ESM (Hektner et al., 2007) is a type of ambulatory assessment (Trull and Ebner-Priemer, 2013) that aims to tap into the day to day experiences of people as they go about their lives. Rather than relying on retrospective reporting, which can be subject to various types of cognitive bias (Schwarz and Sudman, 2012), the ESM involves prompting participants throughout the day to report on their experiences right then and there. An advantage of this methodology is that it yields data with high ecological validity, because the measurements are taken in real-life situations. The ESM is a popular approach in the study of affect, because it can be used to gain a more in-depth or fine-grained understanding of affect dynamics at the individual level. Examples of studies that employed the ESM to investigate affect include Suls et al. (1998) and Koval and Kuppens (2012); an overview of research approaches to affect dynamics is given by Hamaker et al. (2015).

An important aspect of an ESM study design, and what interests us most here, is the signaling or measurement schedule, that determines when and how often measurements are taken. Hektner et al. (2007) distinguished between three general classes of measurement schedules in ESM studies: Event-contingent sampling, interval-contingent sampling, and signal-contingent sampling (also sometimes called random time-based sampling; Bolger et al., 2003). The choice for one or the other measurement schedule in a particular study is theory-driven and can have important implications for the validity of the findings. In this paper, we focus on the data analytic implications of choosing a signal-contingent sampling schedule. This appears to be the most widely used of the three, and as a result, this type of measurement schedule has come to be seen by many researchers as a characteristic feature of ESM research. In this approach, measurements occur after random beeps that are delivered by a signaling device; usually the same device that is used for recording the self-reports. An important advantage is that predictability of the measurements is avoided, increasing ecological validity. The person's waking hours are divided into blocks of time, usually around 90 to 120 min, and then the exact beep time within each block is determined by a random sampling procedure. Typically, the beep time within a block is sampled from a uniform distribution, so that in principle, the beep is equally likely to occur at any moment. However, researchers can impose restrictions, for instance, prohibiting beep intervals shorter than 20 min.

**Figure 1:**
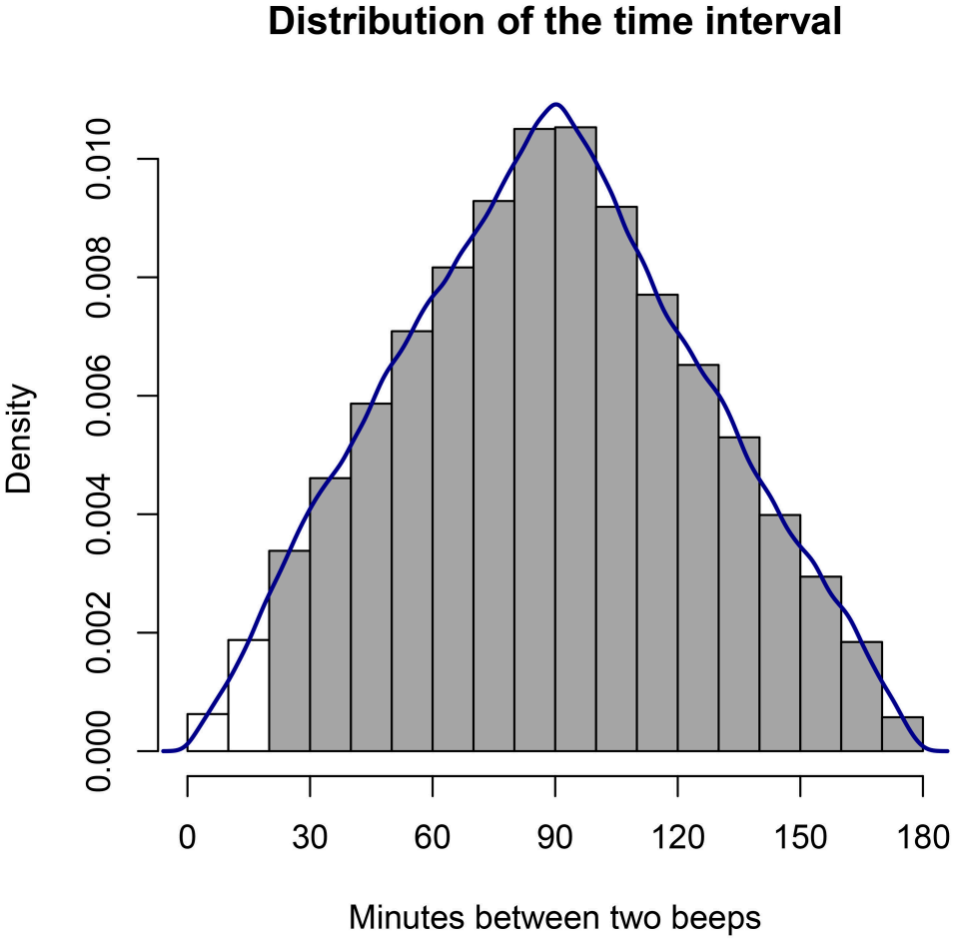
Distribution of the time intervals between consecutive measurements in an ESM study when beeps are uniformly randomly distributed within blocks (here, a block equals 90 min). In practice, it may be desirable to impose a minimum time interval, effectively truncating the distribution.

It is important to see how the intervals between measurements in a signal-contingent sampling scheme will vary. If the probability distribution for the beep *time* within each block is uniform, this implies that the time difference or *interval* between two measurements follows a triangular probability distribution with a lower bound of *a* = 0, an upper bound *b* of two times the length of a block, and a mode *c* equal to the length of one block. For instance, suppose that each block is *l*_*b*_ = 90 min long, and that we start counting at 0; suppose, further, that we ignore any practical limitations on infinitely short intervals. Then the first beep can occur anywhere between 0 and 90 min with equal probability, and the second beep can occur anywhere between 90 and 180 min with equal probability. The triangular distribution for the interval between two consecutive beeps in this example is depicted in Figure 1. Its mean or expected value is defined as $\frac{\left(a+b+c\right)}{3}$, which gives us $\frac{\left(0+2l_{b}+l_{b}\right)}{3}=l_{b}$ or 90 min. The variance of a triangular distribution is given by $\frac{a^{2}+b^{2}+c^{2}-ab-ac-bc}{18}$, which can be simplified in our case to $\frac{3l_{b}^{2}}{18}$, yielding 1,350 min. This distribution is symmetrical and its mode is equal to the mean, but this only applies when even the shortest intervals are possible and allowed. If short intervals are excluded, the distribution becomes truncated and asymmetrical; the mean will be larger than the mode; and the variance will be reduced.

### 2.2. VAR(1) models for ESM data

Data obtained using the ESM can be analyzed in various ways (for instance, the time series data can be aggregated to obtain statistics at the person level), but here we focus on (vector) autoregressive (AR or VAR) modeling, because this is commonly used in research areas like emotion dynamics and psychopathology. In some applications in the literature, researchers used multilevel models, in which the fixed parameters represent population averages and the random effects account for individual differences (e.g., Kuppens et al., 2010; Bringmann et al., 2013). In the current article, we focus on the *N* = 1 case for simplicity.

The VAR model—of which the AR model is a special case for univariate data—is used to model the dependency between consecutive observations in a (multivariate) time series (cf. Hamilton, 1994; Box-Steffensmeier et al., 2014). For instance, participants in an ESM study may have rated the intensity of their affect at each measurement, or they may have reported on multiple specific emotions, behaviors, or thought processes. The term “spill-over” is used to reflect the extent to which the current state (e.g., a person's current emotion) influences the state at a later time. We can estimate the spill-over within a single variable, and in the multivariate case also the spill-over between variables, which is captured in cross-lagged coefficients. The first-order VAR model, denoted as a VAR(1) model (Hamilton, 1994), is given by

(1) $$\begin{array}{l} y_{i}=c+\Phi y_{i-1}+\epsilon_{i}, \end{array}$$

where ***y*_*i*_** is the vector of *K* outcome variables observed at measurement *i*, and **Φ** is a *K* × *K* matrix of regression coefficients. The 1 in the name of the model refers to the assumption that there is no correlation between ***y***_*i*_ and any of ***y***_*i*−2_, ***y***_*i*−3_, ***y***_*i*−4_, … after conditioning on ***y***_*i*−1_. The diagonal elements of **Φ**, denoted as ϕ_11_, ϕ_22_, …ϕ_*KK*_, are the autoregressive parameters, and the off-diagonal elements represent the cross-lagged effects between variables, such that ϕ_12_ is the regression coefficient for predicting *y*_1*i*_ from *y*_2(*i*−1)_. The vectors ***c*** and **ϵ**_*i*_ are the intercepts and the innovations for observation *i*, respectively, and the innovations are assumed to follow a multivariate normal distribution with a zero mean vector and covariance matrix **Σ**_**ϵ**_. This covariance matrix is related to the stationary covariance matrix of the process (**Σ**_**y**_) by

(2) $$\begin{array}{l} \Sigma_{\epsilon }=\Sigma_{y}-\Phi \Sigma_{y}\Phi^{\prime }, \end{array}$$

where **Φ**′ is the matrix transpose of **Φ**.

In the special case of a univariate process, the VAR(1) model reduces to what is known as the AR(1) model. For a VAR(1) process to be stationary, there are restrictions on the parameter **Φ** (Hamilton, 1994), which in the univariate case (that is, in the AR(1) model) simply amount to −1 < ϕ < +1. The stationary mean of an AR(1) process is $\mu_{y}=\frac{c}{1-\phi }$, from which it can be seen that if the intercept (*c*) is zero, so is the mean.

The parameter ϕ in the AR(1) model has been referred to as emotional *inertia* in affect research applications (Suls et al., 1998), because a higher value of ϕ indicates greater predictability in affect, or put differently, more spill-over of affect from one moment to the next. As a descriptive measure of a person's affect dynamics in daily life, inertia appears to be related to personality traits and psychological well-being (cf. Hamaker et al., 2015; Houben et al., 2015). In a different vein, VAR modeling of ESM data plays an important role in the recently developed network approach to psychopathology (Borsboom and Cramer, 2013; Bringmann et al., 2013), where the estimated regression coefficients for different symptoms can be used to construct a dynamic network that provides insight into a person's proneness to, for instance, clinical depression, and the progression of symptoms over time. Two other, recent examples of VAR model applications using ESM data are the empirical illustrations in the methodological studies by Schuurman et al. (2016b) and Schuurman et al. (2016a).

### 2.3. ESM data typically violate a VAR model assumption

The VAR model is a *discrete-time* (DT) model, because it assumes that time proceeds in equal discrete steps between each pair of observations. We can see in Equation (1) that one unit of time is assumed to have passed between each two observations, because ***y*** at measurement *i* is always predicted from ***y*** at measurement *i* − 1 without specifying the amount of time that passed between the measurements. This is quite a restrictive assumption, and it is typically violated in ESM data, even if there are no missing observations.

In most studies using the ESM, measurements are taken over a period of multiple days, resulting in several short time series separated by stretches of nighttime. To analyze such data as if they constituted a single long time series with equal intervals would be inappropriate, as it would ignore that the time interval between the morning's first measurement and the previous night's last measurement is much longer than the time intervals between measurements on the same day (in addition to possible interval length variation within a day). Thus, in the specification of an AR or VAR model for such ESM data, at the very least we would want to prevent the first measurement of 1 day from being regressed on the last measurement of the previous day. To achieve this, we could set the values of the predictor ***y***_*i*−1_ to missing values for those outcomes ***y***_*i*_ which are the first measurements of a day.

A trickier problem for AR and VAR modeling is the within-day timing of measurements in signal-contingent sampling schemes, where measurements are taken at irregular times. Even if the day in such a sampling schedule has been divided up into blocks of equal length, the exact timing of each beep is varied randomly so that the interval lengths will vary. Obviously, then, the resulting data will violate the assumption of equal spacing inherent in AR, VAR and other DT models. It remains to be seen how robust the parameter estimates of interest might be to the violation of this assumption. Since many studies in the literature have relied on DT modeling of unequally spaced ESM data, this is an important question. If the violation of this assumption causes serious bias in the parameters of interest, not only would we have to conclude that DT models are unsuitable for unequally spaced ESM data and that *continuous-time* (CT) models should be used instead, but this would also cast doubt on previous findings.

**Figure 2:**
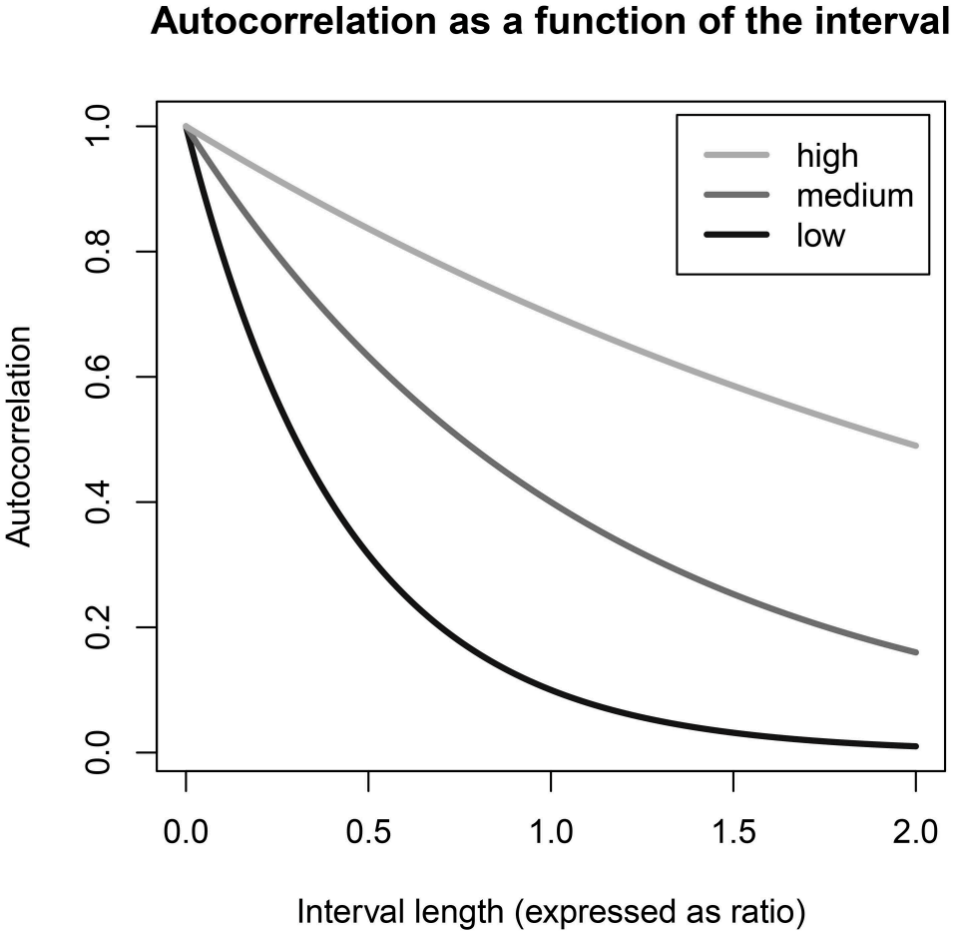
The relationship of the autocorrelation to the (proportionate) interval length Δ, given by ϕ raised to the power of Δ, is illustrated here for cases where the autocorrelation for a one-block interval is 0.1, 0.4, or 0.7, labeled as low, medium, and high ϕ parameters, respectively. The interval length is expressed as a ratio of the block length chosen in the study design, such that an interval of 0.5 is half a block and 2.0 is two blocks; and the depicted relationship holds regardless of what amount of time (e.g., 60 or 120 min) is considered to be “one block.” When the autocorrelation for a one-block interval is low to medium, shorter and longer time intervals in an empirical data set cannot be expected to cancel each other out exactly in the estimation of ϕ.

Since the distribution of the time intervals obtained by random beeps within blocks is (nearly) symmetric, at first glance we may think that there should only be a problem with noise, and not with bias, as it may appear that shorter and longer time intervals will “cancel out” in estimating the **Φ** which applies to the interval length of one block. However, as illustrated in Figure 2, the relationship between the interval length and the true autoregressive coefficient(s) is non-linear, so that shorter and longer time intervals are *not* associated with symmetric increases and decreases in the true autocorrelations. This becomes clear if we iteratively fill in the predicted (determined) part of Equation (1), to make explicit the *assumed* relationship between observations that do not directly follow each other in the measurement sequence. Under the AR(1) model,

$$\begin{array}{l} y_{i}=c+\phi y_{i-1}+\epsilon_{i}, \end{array}$$

and

$$\begin{array}{l} y_{i-1}=c+\phi y_{i-2}+\epsilon_{i-1}. \end{array}$$

Therefore, it follows implicitly that

$$\begin{array}{l} y_{i}=c+\phi (c+\phi y_{i-2}+\epsilon_{i-1})+\epsilon_{i}\\ \;=(c+\phi c)+\phi^{2}y_{i-2}+(\phi \epsilon_{i-1}+\epsilon_{i}), \end{array}$$

where the last part (between parentheses) represent the accumulation of unexplained (innovation) residuals, and where we can see that the expected correlation between *y*_*i*_ and *y*_*i*−2_ is ϕ^2^. Thus, estimating a lagged relationship between consecutive observations, as we do in AR modeling, always implies a non-linearly decaying relationship between observations at longer lags. For any two measurements separated by Δ times the measurement interval, the predicted correlation between *y*_*i*_ and *y*_*i*−Δ_ is ϕ^Δ^, and this also holds in the hypothetical case of observations separated by decimal lags. Therefore, if the true model underlying our data is an AR model, and the measurement intervals are irregular, we should expect that the true correlation between some consecutive measurements is much higher than that between others, and that they are *not* equally close to the true correlation for the *average* time interval.

Consider the plotted line for a “low” autocorrelation, by which we mean a true ϕ coefficient of 0.1 at an interval length of one block, or 90 min. If the data we are using to estimate this parameter has some intervals of 45 min (0.5 block) and some of 135 min (1.5 block), the true autocorrelation for the shorter interval will be 0.1^0.5^ = 0.32, whereas for the longer interval it will be 0.1^1.5^ = 0.03. Thus, while the average of these two interval lengths is one block, the average of the autocorrelation coefficients associated with them is 0.18 instead of the 0.10 which we know is the autocorrelation ϕ for a one-block interval. This illustrates that when the autocorrelation ϕ for a one-block interval is low, shorter intervals in the data set can be expected to exert more influence on the estimation than longer intervals, positively biasing the estimate of ϕ. In contrast, as Figure 2 shows, when the true autocorrelation for a one-block interval is 0.4 or higher, the relationship between the autocorrelation and the time interval is much more linear within the range of interval lengths obtained in a sampling scheme with random beeps within fixed blocks. Therefore, if the true autocorrelation for a one-block interval is expected to be this high, there is little reason to worry about bias in this particular study design.

In the literature, there are many studies involving DT modeling of unequally spaced data where this shortcoming is not addressed. Examples of ways that some researchers did address the issue are adding the length of the time interval as a predictor (e.g., Moberly and Watkins, 2008) or as a moderator (Selig et al., 2012), but this cannot correct for the potential bias in **Φ**, because it does not change the fact that **Φ** is estimated under the false assumption that all the measurements are equally spaced. Another strategy that is useful in some cases is the phantom variable approach, which treats the unequal intervals as a missing data problem (cf. Oud and Voelkle, 2014). However, in ESM research this may not be feasible, since one would need many “missing” observations in between each pair of measurements to account for all the (subtly) different interval lengths that occur in this kind of study design. While it seems feasible to use this approach to at least reduce the random variation in interval lengths, and thus to reduce the potential bias, a more elegant way to handle unequally spaced data is to use CT modeling, which explicitly accounts for the exact time intervals.

## 3. The continuous-time (CT) alternative

Where DT models assume equally spaced data, CT models are more flexible and therefore, more appropriate for unequally spaced ESM data. In a CT model, the actual time intervals are used in the model equations. In this section we present the CT analog to the VAR(1) model, denoted as the CVAR(1) model, and we briefly discuss other considerations from the literature surrounding the choice between DT and CT models.

### 3.1. The CVAR(1) model

The VAR(1) model (and its univariate special case, the AR(1) model) that we presented in section 1 has a CT analog, where a stochastic differential equation is used to model the continuous change (both explained and unexplained) in the process. Using the more general notation for a multivariate case (i.e., for a CVAR(1) model), this equation has the form

(3) $$\begin{array}{l} \frac{dy(t)}{dt}=a+By(t)+G\frac{dW(t)}{dt}, \end{array}$$

where ***a*** is an intercept vector, ***B*** is the drift matrix, ***G*** is the Cholesky triangle of the innovation covariance (also called diffusion) matrix, and ***W***(*t*) is the continuous-time error (Wiener) process (also called Brownian motion). If we assume that the process has a mean of zero (or is analyzed in deviation form, i.e., after mean-centering) we can drop the intercept vector ***a*** to simplify Equation (3). For more details on the differential equation and the steps involved in solving it, we refer the reader to Oud and Delsing (2010), Voelkle et al. (2012), and Oravecz et al. (2009). Here we focus on its solution, which gives us the CVAR(1) model, or the CAR(1) model in the univariate case. Note that the CAR(1) model is also known as the Ornstein-Uhlenbeck process or model (cf. Oravecz et al., 2009).

The CVAR(1) model, which is the solution to Equation (3), effectively predicts the *i*-th observation, which is taken at time *t* and is denoted by ***y***_*t*_*i*__, from the previous observation (denoted by ***y***_*t*_*i*−1__) *and* the time interval Δ_*i*_ = *t*_*i*_ − *t*_*i*−1_ between the two observations. Assuming (for ease of presentation) that all variables are in deviation form, so that the intercepts are all zero, we can write the CVAR(1) model equation as

(4) $$\begin{array}{l} y_{t_{i}}=e^{B\Delta_{i}}y_{t_{i-1}}+\epsilon_{\Delta_{i}}, \end{array}$$

where ***B*** is the drift matrix and is multiplied by the interval length Δ_*i*_, and **ϵ**_Δ_*i*__ is the vector of innovations, with

(5) $$\begin{array}{l} \epsilon_{\Delta_{i}}\sim N(0,\Sigma_{\epsilon_{\Delta_{i}}}). \end{array}$$

The innovation covariance matrix **Σ**_ϵ_Δ_*i*___ for an interval of length Δ_*i*_ is derived by

(6) $$\begin{array}{l} \Sigma_{\epsilon_{\Delta_{i}}}=\Sigma_{y}-(e^{B\Delta_{i}}\Sigma_{y}e^{B^{\prime }\Delta_{i}}). \end{array}$$

In these Equations, $e^{\text{B}\Delta_{i}}$ and $e^{{\text{B}}^{\prime }\Delta_{i}}$ are matrix exponentials, and it can be seen that the covariance matrix for the innovations reduces asymptotically to the stationary covariance matrix **Σ**_*y*_ as the prediction interval Δ_*i*_ increases (since $e^{\text{B}\Delta_{i}}$ then reduces to zero). In the case of a univariate process, *B* is a single value and the matrix exponentials reduce to ordinary exponentials. Conceptually we can see the DT VAR(1) model as a special case of the CVAR(1) model, where time is not explicitly included in the model, but is assumed to only increase in equal, discrete steps, by fixing Δ_*i*_ in the CVAR(1) model to 1 for all *i*^1^. Note that CVAR(1) model parameters can always be transformed into corresponding VAR(1) parameters for any given interval; the DT autoregressive parameter for a time interval of Δ units on the CT model's time scale is given by ϕ = *e*^*B*Δ^. In contrast, VAR(1) parameter estimates can only validly be transformed into corresponding CVAR(1) parameters when the data set, to which the VAR(1) model was fitted, was truly equally spaced.

### 3.2. Considerations in choosing between DT and CT modeling

We now discuss four points of consideration when it comes to the choice between DT and CT models. The first three point to CT modeling as the preferred approach, but the fourth is pragmatic and may (in part) explain the predominance of DT models even for ESM data.

First, an advantage of CT modeling, especially if it becomes widely known and used, is that it allows researchers flexibility in choosing their data collection schemes. Bolger et al. (2003) warned against the danger of making study design choices solely on the basis of data analytic concerns, and this can be applied to situations where DT modeling would call for the collection of equally spaced data. The ecological validity of ESM data is improved by using unpredictable measurements (Hektner et al., 2007), and in some cases unequally spaced measurements are (also) more efficient in terms of gathering information about the underlying process (Voelkle and Oud, 2013).

Second, it has been argued that CT modeling fits more naturally with our theories and hypotheses about how processes are continually evolving, and that the discrete time intervals which become the focus in DT modeling are an artifact of measurement rather than a thereotically meaningful construct (Oud, 2002; Oud and Delsing, 2010). Any ESM researcher who takes measurements at *irregular* intervals has assumed from the outset that the process continues to exist and evolve in between the measurements, so a CT model is a natural choice.

A third consideration in favor of CT modeling is that DT model parameters cannot easily be generalized or compared between studies that use different (average) time lags between measurements, which poses a serious problem for meta-analysis and for theory formation more generally (Oud, 2002; Voelkle et al., 2012; Deboeck and Preacher, 2016). As Deboeck and Preacher show, even the interpretation of parameters within a single DT VAR model easily leads to confusion. In contrast, CT model parameters can always be compared or transformed into corresponding DT parameters for any given time interval of interest.

The fourth point of comparison between DT and CT models is pragmatic: while AR and VAR models (including multilevel extensions) can be implemented in many major statistical software packages, this is not the case for their CT analogs. Recent advances have been made, though, with the publication of the R package *ctsem* for CT structural equation modeling (Driver et al., 2017), which can be used to fit the CAR(1) and CVAR(1) models as well as many other and more complicated models for time series and panel data.

## 4. A simulation study to assess bias in VAR(1) modeling of ESM data

Given that VAR(1) models are frequently used for studying lagged relationships in unequally spaced ESM data, it is important to determine whether any bias that may result is substantial enough to lead to problems in practice, or whether (in some situations) results and substantive conclusions are robust against the violation of the assumption of equal spacing. In this section, we present a simulation study that addressed this question specifically for the context of *N* = 1 AR(1) and VAR(1) modeling of ESM data. Note that, for the sake of brevity, in this section we will use the abbreviations VAR(1) and CVAR(1) when we are referring to both the multivariate and univariate [i.e., the AR(1) and CAR(1)] models.

We compared the performance, in terms of accurate estimation of **Φ**, of VAR(1) and CVAR(1) models, under different specifications. We simulated data under the CVAR(1) model, examining both a univariate and a bivariate case, and assuming that ten measurements are taken per day for either 10 days (giving us a short time series) or 1,000 days (giving us a very long time series). The data were generated using a signal-contingent sampling scheme where the waking hours (15 h) of each day are divided into 10 back-to-back 90-min blocks during which a beep can randomly occur. In a first round of simulations it was assumed that any interval length, including very small intervals > 0, could occur (although very short intervals have a low probability); in a second round of simulations, we put a restriction on the intervals such that they must be at least 15 min long, which is more realistic. By comparing the results, we could get an impression of the bias that may occur in the theoretical “worst case” scenario as well as in a more realistic research scenario. We expected that prohibiting the shortest possible intervals attenuates the bias caused by ignoring the unequal spacing, since the shortest intervals are associated with the largest true autocorrelation, as was illustrated in Figure 2.

### 4.1. Data generation

The simulated data were generated using R (R Core Team, 2016) and the R packages *MASS* (Venables and Ripley, 2002), *matrixcalc* (Novomestky, 2012), and *expm* (Goulet et al., 2015). The general procedure was as follows: First we generated the exact beep times according to a signal-contingent sampling scheme for the specified number of days, measurements per day, and block length. Time was scaled in such a way that a Δ value of 1 corresponded to a block of 90 min, and the minimum interval length was either Δ_*i*_ > 0 or $\Delta_{i}>\frac{1}{6}$, with the latter restriction corresponding to a minimum interval of 15 min between measurements, used in the second round of simulations. In all simulations we assumed that there are ten measurements per day, and that the process continues unobserved throughout the night; each day's first observation was only (very) weakly correlated to the previous day's last observation. The exact time intervals Δ_*i*_ for all pairs of consecutive measurements ***y***_*t*_*i*__, ***y***_*t*_*i*−1__ were calculated by subtracting their exact times *t*_*i*_ and *t*_*i*−1_.

The first observation was randomly drawn from the stationary distribution, that is, from a (multivariate) normal distribution with a zero mean (vector) and (co)variance (matrix) **Σ**_*y*_. The means of the time series were always fixed at 0, and the variances at 1, giving us standardized variables. For each consecutive observation starting at *i* = 2, we used the obtained values of Δ_*i*_ and ***y***_*t*_*i*−1__ together with the specified ***B*** to derive the predicted value(s) of ***y***_*t*_*i*__ according to Equation (4) (which holds for mean-centered variables). To obtain the actual ***y***_*t*_*i*__, we added to this prediction the random innovation term(s) **ϵ**_Δ_*i*__, which were distributed as in Equation (5), with **Σ**_ϵ_Δ_*i*___ derived from Δ_*i*_ and the parameters ***B*** and **Σ**_*y*_, using Equation (6).

#### 4.1.1. Univariate

In the univariate simulations, we used nine different values for the autoregressive parameter ϕ for an interval length of Δ = 1, ranging from 0.1 to 0.9 in increments of 0.1. Although the largest of these ϕ values are less likely in practice, using both small and large values provided us with a complete picture that enabled us to see how the bias depends on the true autocorrelation. Note that, due to the differing scales of ϕ and *B*, a true ϕ of exactly 0 would correspond to an infinitely negative *B*, whereas a ϕ close to its upper bound of 1 would correspond to a *B* close to its upper bound of 0. The values 0.1 to 0.9 for ϕ thus corresponded to values −2.30 to −0.11 for *B* in the CAR(1) model. For each condition we simulated one very long time series with 1,000 days (giving us 10,000 observations) to study the asymptotic performance of the models. In addition, we generated 1,000 shorter time series with 10 days each (giving us 100 observations per time series), to investigate the (variability in) performance for a more realistic sample size.

#### 4.1.2. Bivariate

To ensure that **Φ** and **Σ** in the bivariate simulation had realistic magnitudes for ESM research, we based our choice for these parameters on the fixed-effects estimates reported in Schuurman et al. (2016b), who estimated a multilevel bivariate VAR(1) model for empirical data on Positive Affect and Worrying. We generated standardized variables by first transforming the reported parameter estimates from their study, setting the mean to zero for both variables, and obtaining

$$\begin{array}{l} \Sigma_{y}=\left[\begin{array}{cc} 1 & -0.5155\\ -0.5155 & 1 \end{array}\right] \end{array}$$

for the covariance matrix. The standardized matrix **Φ** (for Δ = 1) was

$$\begin{array}{l} \Phi =\left[\begin{array}{cc} 0.3540 & -0.0482\\ -0.0679 & 0.2770 \end{array}\right], \end{array}$$

which corresponds (approximately) to the drift matrix

$$\begin{array}{l} B=\left[\begin{array}{cc} -1.0541 & -0.1554\\ -0.2188 & -1.3021 \end{array}\right]. \end{array}$$

Using these CVAR(1) parameters, we simulated one very long time series (with 1,000 days) and 1,000 shorter ones (with 10 days each), just as we did in the univariate simulation.

### 4.2. Analysis

All analyses were implemented using the *ctsem* package in R (Driver et al., 2017), because it provides us with a straightforward way of implementing not only the C(V)AR(1) model, but also DT models like the AR(1) and VAR(1) model. Empirical researchers applying an AR(1) or VAR(1) model have many options for the implementation, but in our simulations we wanted to use the same software for all four model specifications. This ensures the comparability of the results across the models, both in terms of parameterization and estimation procedures. Since an analysis in *ctsem* involves specifying the time intervals in the data, we can choose to specify the time intervals in such a way that, for practical purposes, a DT model is obtained (by setting all intervals to the same value). We can also specify the time intervals in such a way that a “compromise” between a DT and CT model is obtained, as will be discussed in more detail below. This allows us to implement models that resemble two common data adjustment strategies in DT modeling of ESM data: removing the nighttime interval from the model, and inserting missing values in the data to reduce the differences in interval lengths. Any differences in performance between our model implementations can be attributed to how they account for the time intervals, as there are no confounding factors like differing estimation algorithms or parameter restrictions.

Each of the simulated data sets was analyzed with four different model specifications, which are illustrated in Table 1; the first three are different DT model implementations, and the fourth is a true CT model. Model implementation (MI) 1 represents the standard VAR(1) model approach, where all information about the interval lengths is ignored, and the data are analyzed as if all time intervals are exactly equal, including the nighttime interval. Of course, this is a crude modeling approach which we would not recommend to any researcher. MI 2 is better in that it takes the longer nighttime interval into account, but it still relies on assuming that all the measurements within days are equally spaced. This approach is quite similar to a common analysis approach for ESM data, where researchers fit VAR(1) models after setting the lagged predictor to a missing value for those cases where the first measurement of a day is predicted; that way, they prevent a day's first measurement from being regressed on the previous day's last one. Our third MI goes one step further, by using an approximation of the true interval lengths up to a certain precision of, in our case, 15 min (or $\frac{1}{6}$th of a block). This MI is interesting because, as discussed in a previous section, one proposed method of dealing with unequal time intervals is to use phantom variables (deliberately added “missing” data points) to at least partly account for timing in a DT model. As a result, differences in interval lengths are accounted for *up to a certain extent*, and this is what we have in our MI 3. This is implemented in *ctsem* by rounding the true time intervals to increments of $\frac{1}{6}$th, as illustrated in Table 1, and using these rounded values as model input instead of the true intervals. Finally, our fourth MI is the true CT model which uses the exact interval lengths between the measurements, and which we expect gives us the least biased estimates of **Φ**. The main question of interest to us is whether MI 3 or even MI 2 approximates this closely enough that we can conclude that a DT VAR(1) model with appropriate cautionary measures—removing the nighttime interval from the analysis, and possibly also inserting missing data—is an acceptable substitute for the CVAR(1) model in practice.

**Table 1:** Illustration of the difference between the true time intervals, which are used in the CT model (MI4 **Δ**_*i*_) and are given in the right-most column, and the assumed time intervals in the other model implementations (MI 1 to MI 3, in worst to best order).

**Block**	**Beep *t*_*i*_**	**MI 1 Δ_*i*_**	**MI 2 Δ_*i*_**	**MI 3 Δ_*i*_**	**MI 4 Δ_*i*_**
Day 1, 1	0.069	-	-	-	-
Day 1, 2	1.818	1	1	1.67	1.75
Day 1, 3	2.943	1	1	1.17	1.12
Day 1, 4	3.269	1	1	0.33	0.33
Day 1, 5	4.169	1	1	1.00	0.90
Day 1, 6	5.034	1	1	0.83	0.86
Day 1, 7	6.179	1	1	1.17	1.14
Day 1, 8	7.642	1	1	1.33	1.46
Day 1, 9	8.023	1	1	0.50	0.38
Day 1, 10	9.008	1	1	1.00	0.99
Day 2, 1	16.393	1	7	7.33	7.38
Day 2, 2	17.814	1	1	1.33	1.42
Day 2, 3	18.376	1	1	0.67	0.56
⋮	⋮	⋮	⋮	⋮	⋮

*Note that the blocks are 90 min each, and the beep time is expressed in blocks, so that a time t_i_ of 9 corresponds to 13.5 h after the start of the study, and a Δ_i_ of 0.5 to a 45-min interval. The average interval between evening and morning measurements is 7 blocks, corresponding to 10.5 h*.

We discuss bias in terms of **Φ** rather than ***B***, because ***B*** has a less straightforward scale, which makes it harder to evaluate the relevance of a given amount of bias^2^. Another reason to focus on **Φ** is that these DT parameters are commonly reported in the literature, and our interest is whether and how much they may be biased because of the timing issue. Thus, we derive the implied **Φ** for an interval length of one block (i.e., 90 min or Δ = 1) from the ***B*** obtained in *ctsem*, and then we compare them with the true **Φ** for that interval.

### 4.3. Results for the univariate data

#### 4.3.1. For one very long time series

**Figure 3:**
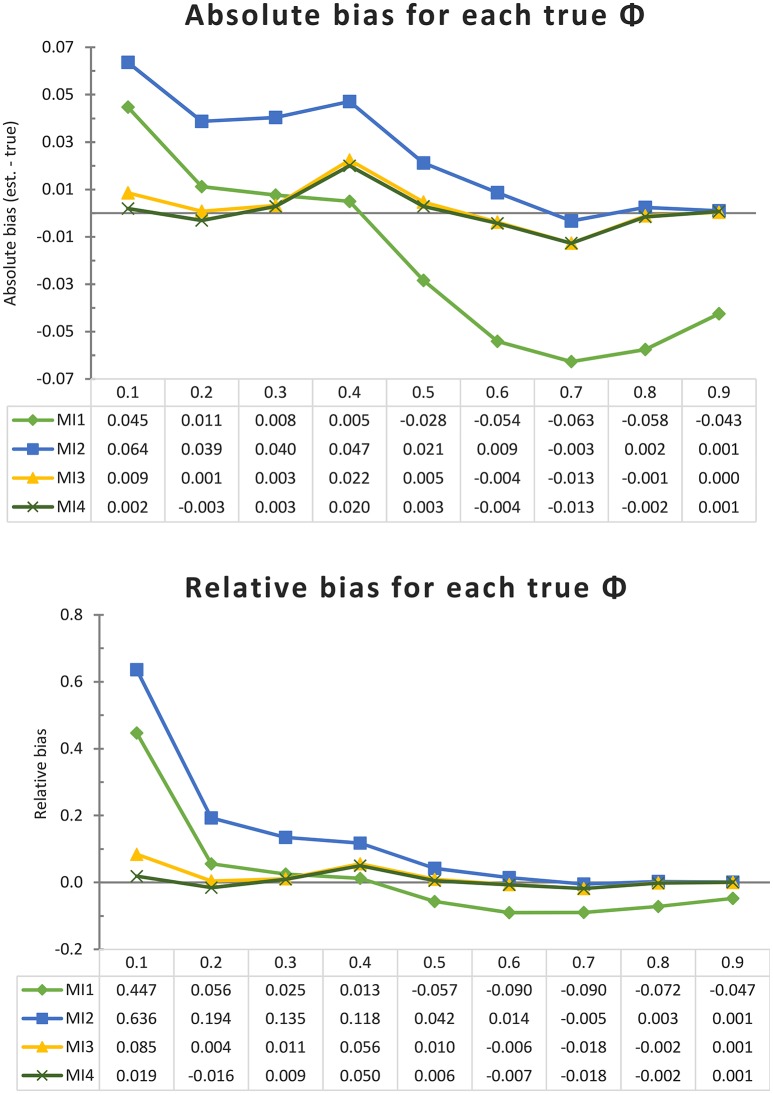
Absolute and relative bias for each model implementation and each true ϕ in the univariate simulation with one very long time series (of 1,000 days) and no minimum interval length. Each line represents a model implementation, and on the X-axis we have the nine different true values of ϕ.

First we consider the results of the simulations allowing all theoretically possible (short) interval lengths, representing the “worst case” scenario where the most potential bias in the DT model is expected. The upper pane of Figure 3 illustrates the absolute bias (estimate—true value) in ϕ (for an interval length of Δ = 1) for each of the four MIs and for each true ϕ; the lower pane illustrates the relative bias, defined as the absolute bias divided by the true parameter size. It can be seen that for 8 out of the 9 parameter values, the CAR(1) model (MI 4) performed better than MI 1 and 2, which resemble two common AR(1) modeling approaches. An important finding is that MI 3 is barely distinguishable from the CAR(1) model, so it appears that adding this amount of “missing” data (allowing interval lengths to differ by increments of one sixth) is sufficient to counter most of the bias resulting from inappropriate handling of interval lengths. Only when the true autocorrelation for an interval of one block is as low as 0.1 does it appear that MI 3 is still slightly affected by bias due to timing misspecification, as it overestimates the autocorrelation by 0.009, while the CAR(1) model (MI 4) is off by only 0.002.

**Figure 4:**
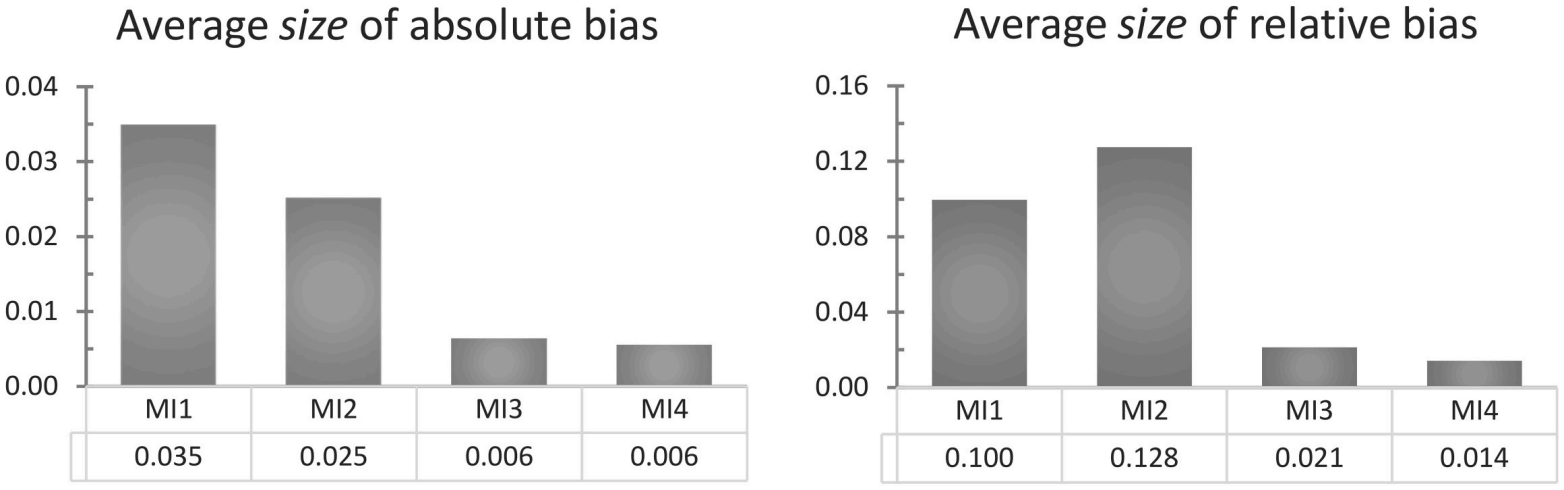
A comparison of the average size of the absolute and relative bias (ignoring its sign) of each model implementation over all true ϕ (from 0.1 to 0.9), in the univariate simulation with one very long time series (of 1,000 days) and no minimum interval length.

To get an overall impression of how the four model implementations perform, we can also average the absolute or relative bias over the nine different true ϕ values, obtaining the results shown in Figure 4. Whether we look at the absolute or relative bias does not change the conclusion that the CAR(1) model and its closest approximation, MI 3, clearly outperform MI 1 and 2, indicating that inappropriately assuming equal time intervals causes positive bias in the autocorrelation estimate, as we expected. Note that the comparison between MI 1 and 2 reverses depending on whether we look at the absolute or the relative bias. In Figure 3 it can be seen that MI 2 suffers from a positive bias for all the true ϕ values of 0.6 and under, congruent with our expectations, but MI 1 suffers less from this positive bias, and is even strongly negatively biased for true ϕ values of 0.5 and higher. To understand why this is, consider first that MI 1 and MI 2 have a source of positive bias in common, namely that they both inappropriately treat measurements within a day as equally spaced. But on top of that, MI 1 adds a second faulty assumption, namely that the nighttime interval is also equally long as all other intervals. This causes an additional bias, which is downwards: Observations which, in reality, are at best very weakly correlated (by ϕ^7^) are treated in this model as if they must be correlated by ϕ, and in our sampling scheme, this applies to 10% of the observations. This negative bias can attenuate or overcompensate for the positive bias that MI 1 has in common with MI 2, and it becomes more pronounced as the true ϕ parameter becomes larger.

#### 4.3.2. For one thousand shorter time series

**Figure 5:**
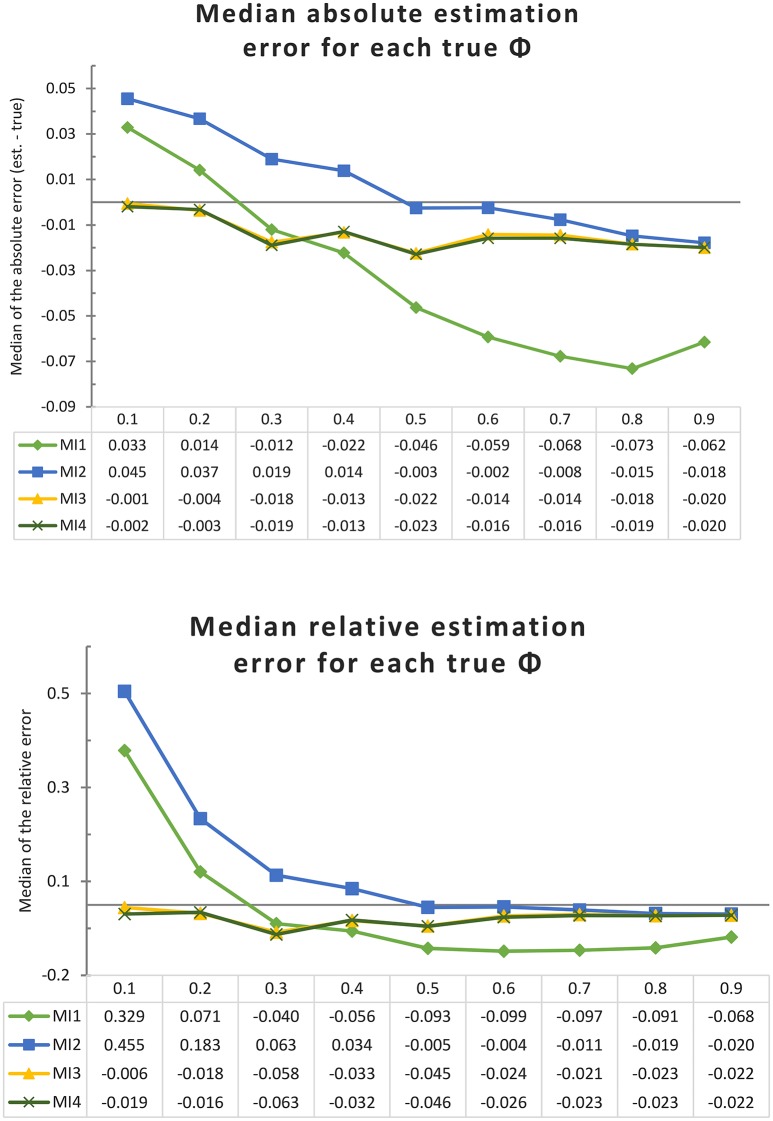
Median absolute and relative estimation error, for each model implementation and true ϕ, over 1,000 shorter univariate time series (of 10 days each), with no minimum interval length. Each line represents a model implementation, and on the X-axis we have the nine different true values of ϕ.

To investigate a more realistic scenario, we also simulated 1,000 shorter time series for each true value of ϕ, with each time series consisting of 10 days (100 observations). Each model implementation was applied to these datasets, and the findings are illustrated in Figure 5. We calculated the deviation between the estimated and true ϕ for each fitted model and then took the median over the 1,000 values as a summary of estimation error or bias. The same was done for the deviation divided by the (absolute) true parameter value, to get an indication of relative rather than absolute bias. We used the median because the distribution of the deviations tends to be skewed when the true parameter ϕ is near its lower (0) or upper (1) bound, making the mean an inappropriate summary statistic.

Comparing the median bias values in Figure 5 with the bias values for the very long time series in Figure 3, we see that the patterns are largely similar. One difference to note is that the median estimation error over one thousand short time series is always negative, regardless of the true parameter value, for the true CT model (MI 4) and for its close approximation MI 3. This is consistent with the known negative bias in the estimated autoregression for shorter time series demonstrated in the literature on both CAR(1) and AR(1) models (cf. Hurwicz, 1950; Marriott and Pope, 1954; Yu, 2012). This also explains why, as the true ϕ increases, MI 1 and especially MI 2 become negatively biased more quickly than they did in the asymptotic case. Judging by the median estimation error, it would appear that MI 2 gives the most accurate estimate of the four implementations whenever the true ϕ is greater than 0.5. This can be explained by the fact that the positive bias particular to MI 2, and the negative bias common to all implementations, cancel each other out to some extent. Given this explanation, the model can hardly be considered a good alternative to the true model MI 4 or its close approximation MI 3; and we can see in Figure 5 that the latter two have the least *relative* median estimation error overall, that is, if we consider the whole range of true ϕ values from 0.1 to 0.9.

### 4.4. Results for the bivariate data

#### 4.4.1. For one very long time series

**Figure 6:**
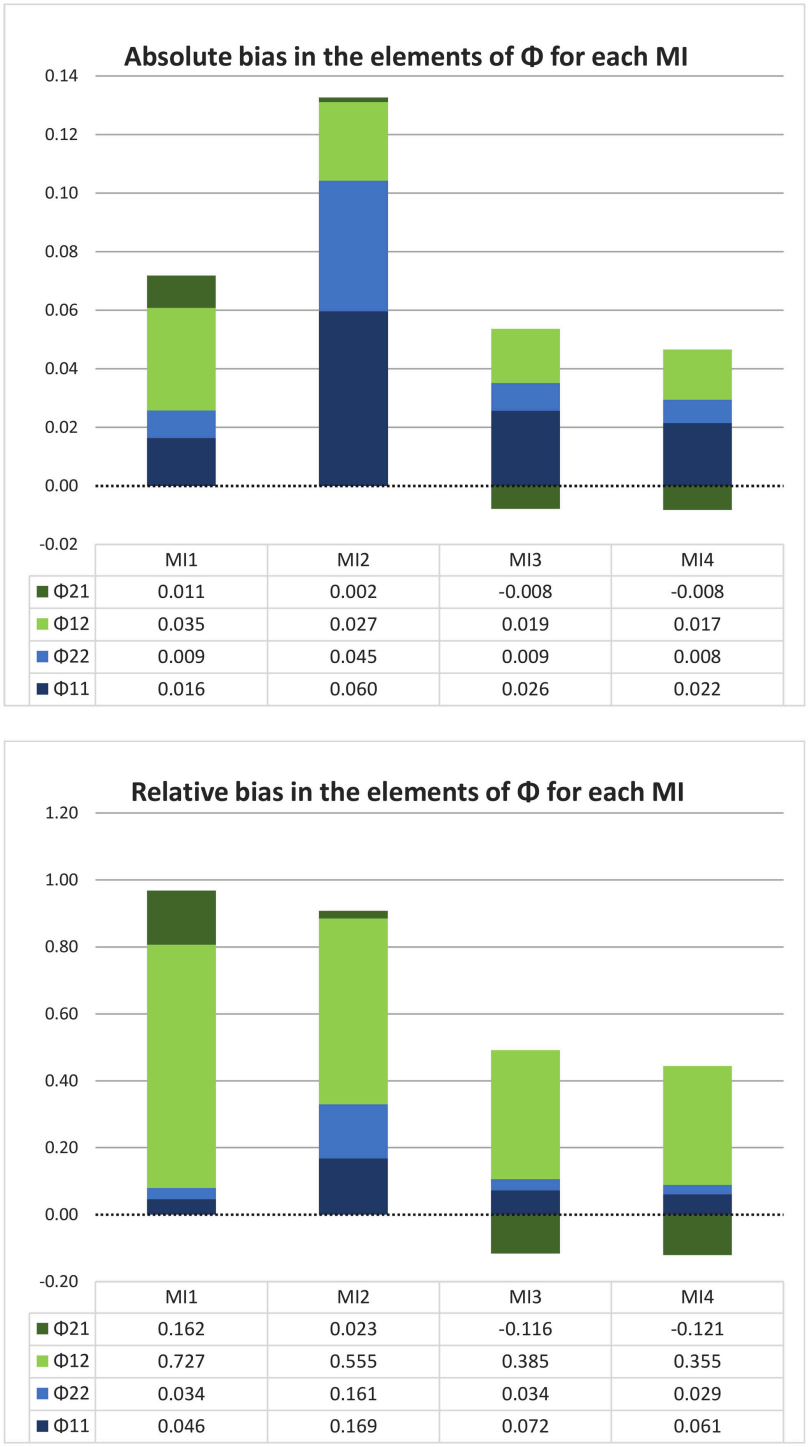
Absolute and relative bias in **Φ** for each model implementation in the bivariate simulation with one very long time series (1,000 days) and no minimum interval length. In this graph, each stacked bar represents a model implementation and each color represents a different element of the matrix **Φ**, with the autocorrelations in blue and the cross-coefficients in green. It can be seen that MI 1 and 2 have a greater total amount of bias (longer bars) than MI 3 and 4, and that only the latter two model implementations have any negative bias (namely, for ϕ_21_). MI 2 stands out particularly because of its large positive bias in both of the autocorrelations.

Our findings for a very long time bivariate time series are illustrated in Figure 6. There it can be seen that MI 1 and 2 suffer from a positive bias for each element in the **Φ** matrix, whereas MI 3 and MI 4 have a negatively biased estimate of one of the off-diagonal elements. The total size of each bar gives an indication of the amount of total bias in the **Φ** matrix, and it is clear that the data-generating, true CT model MI 4 is the least biased overall. MI 2 has the most bias overall, and the reason is that it most strongly overestimates both of the diagonal elements of **Φ**, that is, both the autoregressive coefficients. The fact that MI 1 outperforms MI 2 in this regard can likely be explained by the fact that MI 1 involves several competing sources of bias which can cancel each other out, as discussed above. The bottom pane of the Figure shows the relative bias, which means that the absolute bias in each element of **Φ** is divided by the (absolute value of the) true coefficient to get a better impression of the relevance of the bias. Here it can be seen that MI 1 and 2 differ much less in the *total* amount of *relative* bias, and that MI 1 comes out worse, because MI 1 has a larger absolute bias in the off-diagonal elements of **Φ**, which have smaller true values, so that in a relative sense this bias is worse than the bias of MI 2 in the diagonal elements. MI 4 comes out as the least biased model, overall, whether we look at the absolute or relative bias.

#### 4.4.2. For one thousand shorter time series

**Figure 7:**
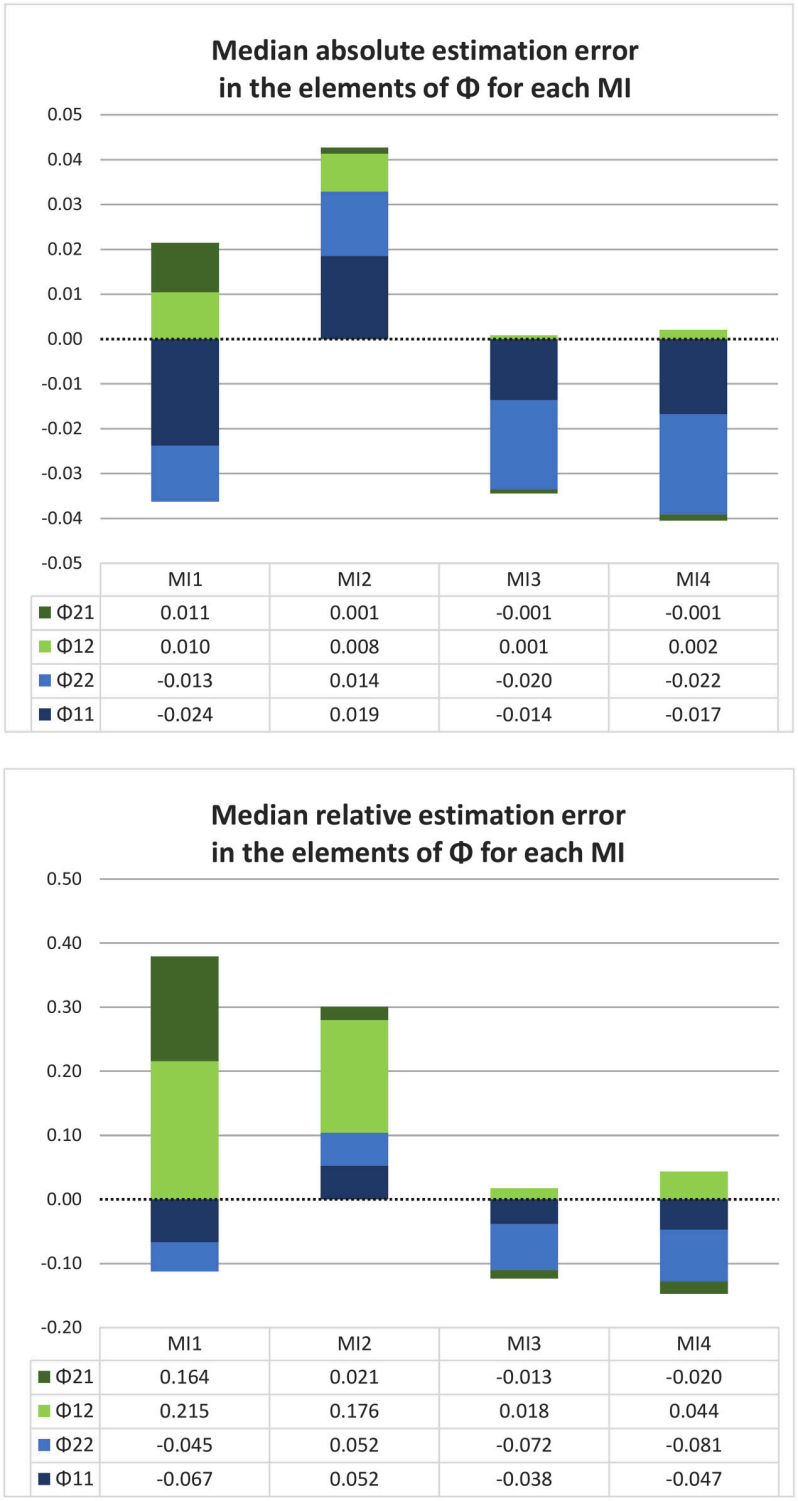
Median absolute and relative estimation error in **Φ** for each model implementation, over one thousand shorter (10-day) bivariate time series, with no minimum interval length. In this graph, each stacked bar represents a model implementation and each color represents a different element of **Φ**. In terms of the total amount of *absolute* estimation error, MI 2 and MI 4 are hard to distinguish and only MI 1 is clearly more biased than the rest; but when we consider the *relative* estimation error, it is clear (from the length of the stacked bars) that MI 3 and MI 4 outperform the other two MIs. MI 2 stands out because it is the only MI that overestimates the autocorrelations.

Turning again to the more realistic case where we have shorter time series, and where we look at the median of the estimation error over one thousand such time series, consider the results for the bivariate data illustrated in Figure 7. Here we see that only for MI 2 the pattern of results is very similar to the asymptotic case, with a positive bias in all four elements of **Φ**, which is the largest for the diagonal (autoregressive) coefficients. For the other three model implementations, however, we note that the results for shorter time series are quite different from those for the asymptotic case. The median estimation error in both of the diagonal elements of **Φ** is now negative for MI 1, 3 and 4, and the positive bias for ϕ_12_ has virtually disappeared for MI 3 and 4. Their negative bias in ϕ_21_ is similarly diminished, but both implementations show a large negative estimation error for the diagonal elements, which were actually overestimated in the asymptotic case. We note that this pattern of results is consistent with what we found in the univariate case, since a negative bias in estimated autoregressive coefficients is to be expected in both DT and CT models for shorter time series. The size of the underestimation in ϕ_11_ and ϕ_22_ for MI 3 and 4 in the short time series is also consistent with our findings for the univariate simulation with short time series, where a median estimation error roughly between −0.01 and −0.02 was found for true **Φ** values between 0.4 and 0.3 (recall that the values of ϕ_11_ and ϕ_22_ in the bivariate simulation are 0.354 and 0.277, respectively). Furthermore, in both of the simulations we see that MI 2 is the only implementation that does not show this well-known negative bias in estimated autoregressive coefficients in shorter time series, but as we discussed above, this does not justify the conclusion that it is to be preferred. What is consistent between the asymptotic simulation and the one with shorter time series is that MI 4 and MI 3 clearly have less total estimation error in **Φ** than MI 1 and 2, and that MI 4 has the least estimation error overall (although the difference between MI 3 and 4 is very small).

### 4.5. The effect of prohibiting intervals shorter than 1/6th of a block (or 15 min)

All the above simulations were repeated while prohibiting measurement intervals shorter than 15 min (one sixth of a block), to investigate more realistic circumstances. To save space, the bias results under this restriction are provided in the Appendix; and here we only summarize the most noteworthy results and our conclusion.

In the univariate case, the positive bias in the autoregressive parameter in MI 1 and 2 is somewhat ameliorated by prohibiting the shortest measurement intervals, as we expected; however, this effect is only seen for the smallest true ϕ (of 0.1). This finding was the same for the asymptotic case as for shorter time series. No such benefit of prohibiting short time intervals was consistently observed when the true ϕ was larger.

In the bivariate simulations, we find that the total amount of bias in **Φ** for MI 1 and MI 2 is highly similar to what we found while allowing short intervals, so that there is no clear advantage to prohibiting short intervals. A surprising result is that in MI 3 and MI 4 the cross-coefficient ϕ_12_ is more strongly (positively) biased when short intervals are prohibited, and in the specific case where we have shorter time series, ϕ_21_ is also biased more heavily (albeit negatively) when short intervals are prohibited. Thus, it seems that, especially in the realistic case of shorter time series, bivariate VAR(1) model estimates do not necessarily become less biased when short time intervals are prohibited, and CVAR(1) model estimates may even become more biased because of it. An explanation may be that short intervals can be highly informative for estimation, depending on the true parameter size(s). Under these simulation conditions, MI 3 and MI 4 no longer even seem preferable to MI 1 and MI 2 in terms of the total amount of (absolute or relative) median estimation error, but this is *not* because MI 1 and MI 2 have become less biased, as we would have expected; it is only because MI 3 and MI 4 have become more biased.

In summary, it is not clear that prohibiting intervals shorter than 1/6th of a block is an effective way of reducing the bias in the DT models, except in the specific case of a univariate process where it is expected that the true autocorrelation at a lag of 1 is very small (ϕ ≤ 0.1).

## 5. Conclusion

Our simulations showed that the bias caused by misspecification of the timing of the measurements depends to a large degree on the true parameters of the process, in such a way that it appears to be most relevant when the true effects for an interval of one block are small. As discussed in the literature, both the AR(1) and CAR(1) model suffer from a negative bias in the autoregressive parameter when short time series are analyzed (Hurwicz, 1950; Marriott and Pope, 1954; Yu, 2012), but this bias tends to be small relative to the true parameter size. In our simulations we found that the cruder model implementations MI 1 and MI 2, which correspond to common DT applications of AR(1) models in the literature, can result in much more severe bias. For small autocorrelation values (< 0.3), which are commonly reported in the literature, MI 2 can result in a bias as large as 60% of the true parameter size. The fact that MI 1 and MI 2 are differentially biased for different true values of **Φ** also seems problematic given that in practice, researchers often aim to compare the **Φ** coefficients for different persons' time series (or to predict them from person-level covariates). MI 3 and MI 4 appear to be more consistent in both the direction and the absolute size of the bias.

The true model, MI 4, and its close approximation MI 3 typically outperformed the others in terms of overall absolute or relative bias in the **Φ** parameters. This indicates that, in addition to theoretical considerations in favor of CT modeling approaches, they are advantageous in terms of reducing bias in **Φ** when analyzing unequally spaced ESM data. However, since the results for MI 3 and MI 4 were barely distinguishable in our simulations, it seems that, for practical purposes, an approximation of a CT model may be as good as the real thing, under the conditions considered in the present study. In our simulations the specification of MI 3 was such that an interval of one block in the original data is divided into six blocks in the analysis. If the blocks were divided into fewer parts, so that the true interval lengths were approximated less closely, the approach would likely perform less well.

## 6. Empirical illustration

We now analyze empirical data concerning the positive affect of three adolescents, to investigate how DT vs. CT modeling of unequally spaced ESM data can play out in practice. AR(1) models are often used for the analysis of affect data, to investigate differences between individuals in their autocorrelation, which is an indicator of affect regulation (and is often called *inertia* in this context). Therefore, this analysis investigates how the substantive findings in this line of research might differ when CT modeling is used instead of DT modeling, to gauge the practical relevance of the choice between these approaches.

### 6.1. The data

The data that we will analyze resulted from the first wave of ESM data collection in a larger study that was designed to detect at-risk mood profiles related to depression in adolescents. As part of this study, ESM questionnaires were filled out by 244 adolescents (aged 12 to 16) from a high school in the Netherlands, after both the adolescents and their parents had given active informed consent. The study was approved by the ethics committee of the Faculty of Social Sciences at Utrecht University.

The participants filled out the ESM questionnaires throughout the day, including during school hours, as the school administrators had agreed that students were allowed to do so during lessons, if necessary. The questionnaires were delivered on the adolescents' own smartphones, using the mobile app Mypanel. The first wave of ESM measurement lasted for 7 consecutive days, and on each day a maximum of eight measurements were taken between 8 AM and 10 PM. A morning measurement occurred between 8 and 10 AM; an evening measurement between 8 and 10 PM; and there were six measurements between 10 AM and 8 PM. A signal-contingent sampling scheme was used, and each beep was programmed to occur randomly within each block, without imposing a minimum interval length. The participants were instructed to respond to a beep as quickly as possible, but they could respond later, if necessary (in which case, they were instructed to report their affect *at the time of responding*, not their affect at the beep time). The six questionnaires during the day had to be filled out within 1.5 h after the beep; the morning questionnaire within 2 h; and the evening questionnaire within 4 h. The purpose behind this flexibility was to enable all participants to fill out all questionnaires even if they had busy schedules and activities that they could not interrupt. Filling out a questionnaire took approximately 1–2 min.

**Figure 8:**
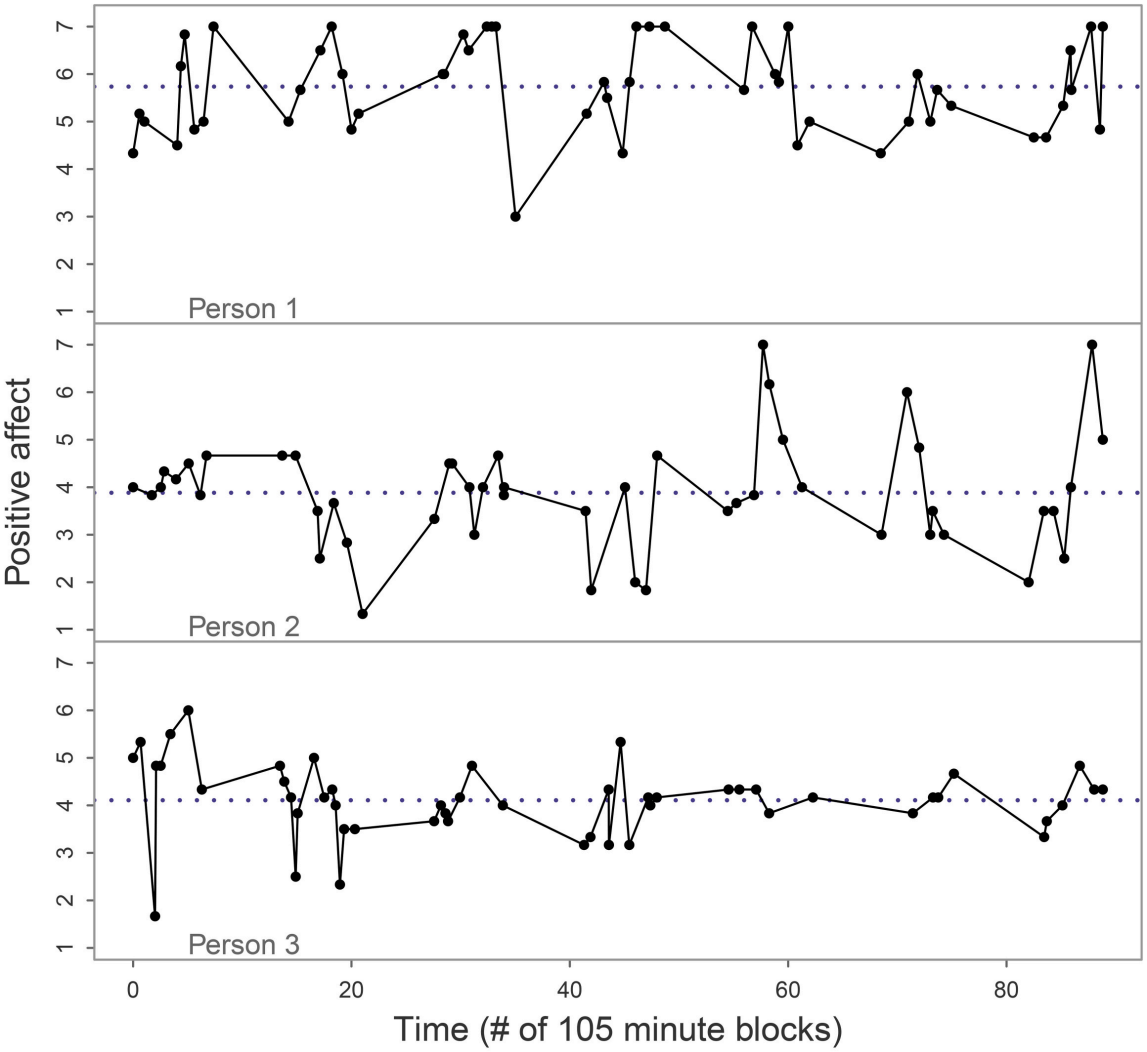
Positive affect data for the three adolescents in our empirical analysis. The dots indicate the observations and reveal that they were spaced irregularly, sometimes following each other quickly. Note that the software recorded the times that the questionnaires were filled out (thus matching the affect that the respondent reported), not the times of the beeps. The dotted line represents the person's mean of PA.

A scale score for the adolescent's positive affect (PA) was obtained by averaging over six specific items, namely feeling relaxed, satisfied, confident, cheerful, energetic, and enthusiastic. Each item was measured using a 7-point scale, where 1 indicated that the respondent did *not* feel that, 4 indicated that they *moderately* felt that, and 7 indicated they *definitely* felt that. The reliability for the PA scale in the first ESM wave of the study was high with a Cronbach's alpha of 0.928 based on 6,213 available data points. For our illustration, we analyze the PA scores of three adolescents who were selected as follows: First, we selected the participants who had completed at least 50 ESM measurements, and then we visually inspected the time series of PA for these nine participants to determine whether they were suitable for (C)AR(1) modeling, given its assumptions. Six of these time series were affected by skewness, ceiling effects, or apparent shifts over time, but the other three looked free of trend, skewness or outliers. By selecting the three time series without (gross) violations of other model assumptions, we could focus on the effect of ignoring the violation of the assumption of equal spacing in the AR(1) model, rather than conflating multiple issues. The time series for the selected adolescents are shown in Figure 8, where the measurement time is expressed in units of 105 min since the start of measurement for that person. Note that the time variable reflects the time at which the respondents *completed* a questionnaire, not the time that a beep was delivered; and if the person did not initially hear the beep or was not able to respond immediately, they might respond a bit later than the beep time. The stored data thus reflect both the time and the affect at the moment of responding. As a consequence, short intervals between observations occur more frequently in these data (namely, when participants had not yet responded to a beep until it was almost time for the next one) than they did in our simulations. The average measurement interval was approximately 105 min.

### 6.2. Analysis

The PA data for each adolescent was analyzed in four ways. Using *ctsem*, we fit a CAR(1) model, and for the purpose of comparison we also fitted MI 3 and 2 in the same way as we did in our simulations. In addition, we used the lm() function in R (R Core Team, 2016) to implement an AR(1) model under the same principles as MI 2. The analysis in lm() is a more likely choice for researchers using DT models in practice: researchers would not use *ctsem* unless they wanted to fit CT models. The CAR(1) model, by definition, uses the exact recorded times of all responses, while the AR(1) model using lm() and MI 2 in *ctsem* only account for the nighttime, not for the random differences in interval lengths between measurements on the same day; and MI 3 accounts for the measurement times up to a precision of 1/6th block (1/6th of 105 min), just as in our simulations.

Each person's data were mean-centered in advance, to aid model convergence (as recommended in Driver et al., 2017). For the AR(1) model in lm(), we created a lagged predictor with the slide() function from the *DataCombine* R package (Gandrud, 2016), ensuring that the lagged predictor was assigned a missing value (N/A) for each first observation of a day. In this way, we prevent treating the nighttime interval als comparable to within-day intervals, because we do not regress 1 day's first observation on the previous day's last one. For the *ctsem* CAR(1) model (MI 4), time was scaled in such a way that Δ_*t*_ = 1 corresponded to a 105-min (or 1.75 h) interval, which was the intended average length of the blocks in the measurement schedule in the study. Because time was scaled in this same way for all three persons, the results for the parameter ϕ at a lag of 1 for the CAR(1) model have the same meaning for all three persons. This is not the case, however, for the AR(1) model estimates obtained using lm(): those reflect the ϕ for a specific person under the assumption that Δ_*t*_ = 1 reflects the time interval between their measurements (within a given day), ignoring potential differences between the three persons in their actual *average* time intervals, as well as variation in the individual intervals for a given person. The AR(1) model (MI 2) estimated with *ctsem* is comparable to the AR(1) model estimated with lm() in that it assumes that the measurements within a day are equally spaced.

We compare the estimated DT-parameter ϕ for all three models and persons, and use the 95% confidence interval (CI) as an indication of the significance of the autocorrelation. For the AR(1) model estimated with lm(), the 95% CI is automatically obtained as part of the output; for the models estimated with *ctsem*, we can obtain a 95% CI for the autocorrelation for any lag (interval length) that occurred in the input data. That means that for MI 2 and 3 in *ctsem*, we can obtain a 95% CI for ϕ at a lag of exactly 1.75 h, similar to what we get for the AR(1) model in lm(), since a lag of 1.75 h or Δ = 1 occurs in the input data: In MI 2 it is assumed to be the lag between *all* consecutive measurements within a day, and in MI 3 this value occurs frequently because the true Δ values are rounded to a precision of 1/6. For the CAR(1) model (MI 4) we can only obtain *approximate* 95% CIs for the ϕ at a lag of Δ = 1 or 1.75 h, because the true interval lengths closest to 1.75 h that actually occurred in the data for the three persons were 1.742, 1.749, and 1.727 h, respectively.

### 6.3. Results

The model estimates are given in Table 2, together with some data descriptives that are relevant for interpreting the models. As can be seen in the Table, both the median and the mean of the interval length between consecutive measurements on the same day were not exactly equal for the three adolescents. Person 2 has somewhat longer measurement intervals, on average, than was to be expected under the beep schedule; but as we noted, participants were able to respond some time after the beep if necessary (e.g., if they had not heard it or if they were unable to respond immediately), which increases the variability of the intervals. For the CAR(1) model, this poses no problem whatsoever, since the exact measurement times are used in the model and we can estimate the ϕ that applies to an interval of 1.75 h for all three adolescents. For the AR(1) model, however, differences between persons in the average interval length may mean that a comparison of their estimated ϕ coefficients is less than perfectly valid, as their coefficients would not apply to the exact same time lag.

**Table 2:** Data characteristics and parameter estimates under four model implementations for the PA of the three adolescents in our empirical example.

	**Person 1**	**Person 2**	**Person 3**
**Data characteristics**			
Median within-day interval (h)	1.495	1.811	1.483
Mean within-day interval (h)	1.773	1.906	1.709
Number of observations	53	50	51
**CAR(1) model (MI 4) in** ***ctsem***			
Autocorr ϕ for Δ = 1.75 hr	0.05	0.39	0
± 95% CI of ϕ	[±0, 0.36]	[0.12, 0.64]	[0, 0]
Stat. var. $\sigma_{y}^{2}$	0.98	1.41	0.61
**Approximation (MI 3) of the CAR(1) model, in** ***ctsem***			
Autocorr ϕ for Δ = 1.75 hr	0.10	0.39	0
95% CI of ϕ	[±0, 0.42]	[0.11, 0.64]	[0, 0]
Stat. var. $\sigma_{y}^{2}$	0.97	1.42	0.61
**AR(1) model (MI 2) in** ***ctsem***, **accounting for nighttime intervals**			
Autocorrelation ϕ	0.19	0.35	0.05
95% CI of ϕ	[±0, 0.50]	[0.07, 0.61]	[±0, 0.33]
Stat. var. $\sigma_{y}^{2}$	0.94	1.36	0.61
**AR(1) model with** ***lm()***, **removing nighttime intervals**			
Autocorrelation ϕ	0.19	0.37	0.06
95% CI of ϕ	[−0.13, 0.52]	[0.06, 0.67]	[−0.24, 0.35]
Stat. var. $\sigma_{y}^{2}$	0.99	1.54	0.66

*Note that exact zeros reported in the table represent values too small for the numerical precision of the software. The 95% CIs for the CAR(1) model are approximations, based on observed lags as close to 1.75 h as possible*.

The results from the CAR(1) model, shown in Table 2, indicate that only person 2 had substantial autocorrelation at a lag of 1.75 h, with an estimated ϕ of 0.39 and an approximate 95% CI that lies far away from zero. It is important to note that in a CAR(1) process, negative autocorrelations are impossible, which means that the 95% CI of ϕ can never include negative values and can only approach 0 more and more closely. In the case of person 2 it is far enough removed from the zero bound to conclude that the autocorrelation is significant, but for persons 1 and 3 the 95% CI was very close to zero, in addition to the point estimates of ϕ being much smaller (0.05 and 0, respectively). For person 3 we reported exact zeros because the software returned them; the values are too minute for the numerical precision. Even the upper bound of the 95% CI for person 3 was extremely close to zero, which clearly indicates a lack of autocorrelation.

Comparing the results of the CAR(1) model with those of its approximation MI 3, the only noteworthy change we see is that the estimated ϕ for person 1 becomes larger (0.10 instead of 0.05) when the interval lengths used in the model are less precise; the lower bound of the 95% CI, however, remains close to zero. Switching to the AR(1) model implementation in *ctsem* (MI 2), the first thing to note is that the overall conclusions remain the same: persons 1 and 3 do not have significant autocorrelation, but person 2 does, and the estimated ϕ of 0.35 for person 2 is quite close to the estimate of 0.39 from the CAR(1) model. The point estimates of ϕ for persons 1 and 3, especially the former, are larger than under the CAR(1) model or its approximation MI 3, and the upper bounds of the 95% CIs have shifted upwards quite a bit, but the lower bounds of the 95% CIs are still so close to zero that the autocorrelation should be interpreted as non-significant. The only way that substantive differences in our conclusions from the different models might arise, then, is if we were to use or interpret the point estimates of ϕ without considering their (non-)significance; for instance, if we were to use covariates to predict differences in ϕ, as is common in multilevel analysis.

When we switch to a true DT AR(1) model in *lm()*, the conclusions again remain unaffected, and the point estimates of ϕ are highly similar between the AR(1) model and MI 2 in *ctsem*. One point of difference is that the lower bounds of the 95% CIs for the non-significant autocorrelations are negative values in the AR(1) model, because a true (DT) AR(1) model does not restrict ϕ to be positive in the way that the CAR(1) model (and thus any *ctsem* model implementation, including MI 2 and 3), does. Although a 95% CI for ϕ that can include negative values might be seen as advantageous for the purpose of significance testing, the possibility of a negative ϕ is inconsistent with the assumption that the process unfolds continuously over time. One could argue that if researchers decide to measure at irregular intervals, they have committed themselves to that assumption from the outset.

Overall, it is reassuring that the significance of the parameters comes out the same between the AR(1) and CAR(1) models for these three time series. However, a point of concern is the change in the point estimate of ϕ for person 1, which was 0.05 in the CAR(1) model, 0.10 in the approximation of the CAR(1) model (MI 3) and 0.19 in both of the AR(1) model implementations. Here we cannot simply assume that the CAR(1) model estimate must be right, because in our simulations we found that even the true model can be biased, and indeed, in some cases it can be biased more severely than cruder models which compensate one source of bias with another, depending on the true parameter size. Based on our simulation findings and on the literature regarding bias in AR(1) and CAR(1) models (Hurwicz, 1950; Marriott and Pope, 1954; Yu, 2012), it may be expected that the CAR(1) model estimate of 0.05 suffers from some negative bias due to the small number of observations: In our simulations we noted a consistent negative bias for time series of one hundred observations, and here we have only 53 observations, so a more severe bias may be expected. This bias should also affect the AR(1) model, but there it may be ameliorated by a positive bias due to ignoring the true length of the measurement intervals. We found that this positive bias is mostly applicable to true parameters of 0.1 (or smaller), so it seems especially relevant for this empirical case, but we can only guess to what extent this bias occurred in this specific empirical application. Since our simulations indicated that, overall, the bias for the CAR(1) model (as well as for MI 3) is smaller than that for the AR(1) model (whether MI 1 or MI 2) and less dependent on the true parameter value, it seems reasonable to put more stock in the CAR(1) model and assume that the estimate of 0.05 is closer to the truth than the estimate of 0.19 under the AR(1) model.

## 7. Discussion

In this article we set out to investigate the practical relevance of a known but seemingly ignored theoretical issue, namely that unequally spaced ESM data violates an assumption of DT models like the AR(1) and VAR(1) model. We discussed why bias in the parameters of interest is to be expected when measurement timing is not taken into account properly, and why CT approaches like the CAR(1) and CVAR(1) models can handle unequally spaced ESM data without any such issues. While we noted that there are several reasons to favor CT models over DT models in general, many previous research findings are based on DT models. Furthermore, AR(1) and VAR(1) will likely continue to be used for ESM data, because they are easy to implement in many statistical software packages (even in the case of multilevel model extensions). Indeed, AR(1) or VAR(1) models are a popular approach to investigating individual differences in autoregressive parameters, e.g., in the context of research into *emotional inertia* or *dynamic network* approaches to psychopathology.

Our simulations with univariate time series confirmed that AR(1) modeling of unequally spaced ESM data may lead to overestimation when the true autocorrelation is (very) small. While the absolute size of the bias was small in many cases in our simulation, and it may seem negligible, the bias can be large relative to the true parameter size, so it should not be ignored. The CAR(1) model was less biased, overall, and more consistent in the size and direction of its bias, than the two AR(1) model implementations that we considered. In the bivariate case, a CVAR(1) model also had less total bias than a VAR(1) model. Furthermore, we found that a close approximation of a CAR(1) or CVAR(1) model, such as could be obtained by inserting “missing values” to reduce the measurement intervals to a finer scale, can be an adequate substitute for the true CAR(1) or CVAR(1) model in terms of its accuracy, at least in all the circumstances we studied: The models were often barely distinguishable in terms of their bias.

We note that all of the models had some estimation error, especially in the case of shorter time series, where they are affected by a negative bias that is known from the literature (cf. Hurwicz, 1950; Marriott and Pope, 1954; Yu, 2012). Paradoxical situations can occur when this negative bias cancels out with the positive bias in a DT model for unequally spaced data, so that it can appear to be a better model, when actually it is compounding multiple sources of bias with unpredictable and highly variable results. In practice there is probably always *some* bias, but a serious issue with the DT models for unequally spaced data is that both the size and the direction of the bias tend to depend strongly on the true parameter. When the interest is in comparing estimated parameters for different persons, a differential bias in their estimates poses a threat to the validity of the analysis.

When a certain minimum time interval is required to pass between consecutive measurements, the bias in a DT model may in some cases be ameliorated, but in other cases it makes little difference or even worsens the estimation of CT model parameters. Having a few very short intervals in the data can be both an advantage or disadvantage depending on whether a CT or DT model is used, and on the true coefficients. In multivariate processes the best observation interval for one effect is dependent on all the other effects, so that we cannot give a general recommendation concerning minimum measurement intervals. In practice, other considerations such as software limitations, respondent burden or ecological validity should factor into this decision.

We recommend that researchers working with unequally spaced ESM data make use of CT models whenever possible. These models provide a natural match with the assumptions and hypotheses involved in observing a process at irregular intervals throughout the day, and they allow researchers more freedom to interpret, compare and generalize their findings, both within or across studies, because of how their estimated parameters can be transformed to DT parameters for any time interval of interest. In addition, the risk of bias arising from unequal measurement intervals in the data is wholly avoided when CT models are used. We consider this to be particularly relevant for studies involving multiple persons where it is crucial that the ordering of the persons' estimated parameters not be affected by differential bias. While DT model implementations that approximate CT models (by adding missing values) can be an acceptable substitute in terms of avoiding bias, they are less appropriate when there are differences in the average measurement intervals of participants; and designing a study to prevent such differences may not always be feasible.

In this paper we focused on *N* = 1 (vector) autoregressive models, but multilevel extensions of these models are in demand as an approach to studying interpersonal differences in dynamics. Multilevel DT AR(1) and VAR(1) models have seen multiple applications, but up until recently there were no ready made solutions in statistical software for implementing multilevel CAR(1) or CVAR(1) models. This has changed now that version 2.3.1 of the *ctsem* R package incorporates a Bayesian routine for estimating CT models with random effects in all parameters (Driver and Voelkle, 2017). In addition, version 8 of Mplus includes Bayesian estimation for various (multilevel) time series models, and it can be used to approach CT modeling by inserting missing values (Asparouhov et al., submitted). In conclusion, then, the door is open for a transition toward new default approaches in dynamic modeling of ESM data, where the measurement schedule is carefully taken into account and the data is used to its full advantage.

## Author contributions

EH conceived of the study; MV contributed to the study design; SdH performed the research and wrote the paper; LK provided the empirical data; and EH, MV, and LK all provided feedback on the analyses and the manuscript.

### Conflict of interest statement

The authors declare that the research was conducted in the absence of any commercial or financial relationships that could be construed as a potential conflict of interest.

## Footnotes

^1^An additional difference between the DT and CT models is that the DT AR(1) / VAR(1) models allow for negative autocorrelations, resulting in a process that tends to oscillate around its mean after each discrete unit of time; such a process cannot be obtained from a CAR(1) / CVAR(1) model. There are, however, other CT models that can represent various sorts of oscillating processes which have no corresponding DT model representation.

^2^Consider, for instance, that *B* in a CAR(1) model can range from −∞ to 0, and that the difference between −1 and −0.1 is more meaningful than that between −100 and −99.1 or that between −100 and −10.
