# Supplementary material for: Discrete- vs. Continuous-Time Modeling of Unequally Spaced Experience Sampling Method Data
Source: Front Psychol. 2017 Oct 20;8:1849. doi: 10.3389/fpsyg.2017.01849 (PMC5655034; doi:10.3389/fpsyg.2017.01849)
Supplement: Supplementary file 1 [file DataSheet1.pdf]

Appendix

Simulation results when intervals shorter than 1/6th of a block were prohibited

Figure A1. Absolute and relative bias for each model implementation and each true φ in the univariate simulation with one very long time series and a minimum interval length of 15 minutes (1/6th of a block). Each line represents a model implementation, and on the X-axis we have the true values of φ.

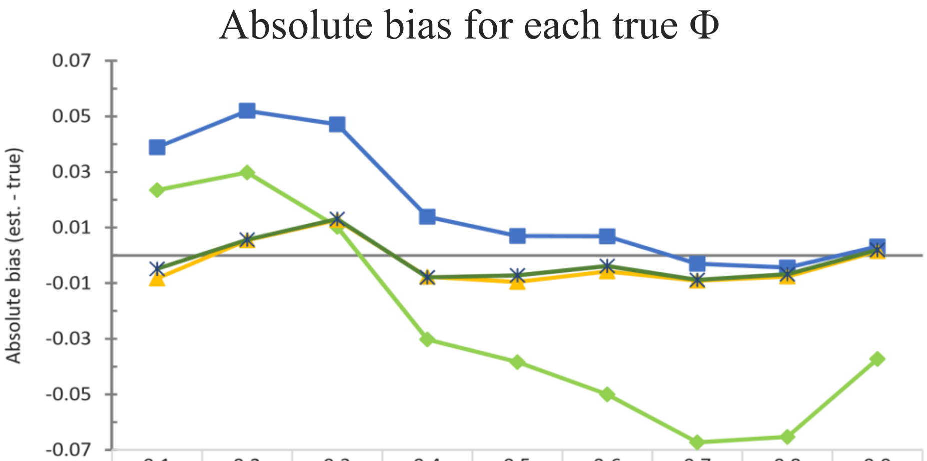

	0.1	0.2	0.3	0.4	0.5	0.6	0.7	0.8	0.9
MI1	0.023	0.030	0.010	-0.030	-0.038	-0.050	-0.067	-0.065	-0.037
MI2	0.039	0.052	0.047	0.014	0.007	0.007	-0.003	-0.004	0.003
MI3	-0.008	0.005	0.013	-0.008	-0.010	-0.006	-0.009	-0.008	0.001
MI4	-0.005	0.006	0.013	-0.008	-0.007	-0.004	-0.009	-0.007	0.002

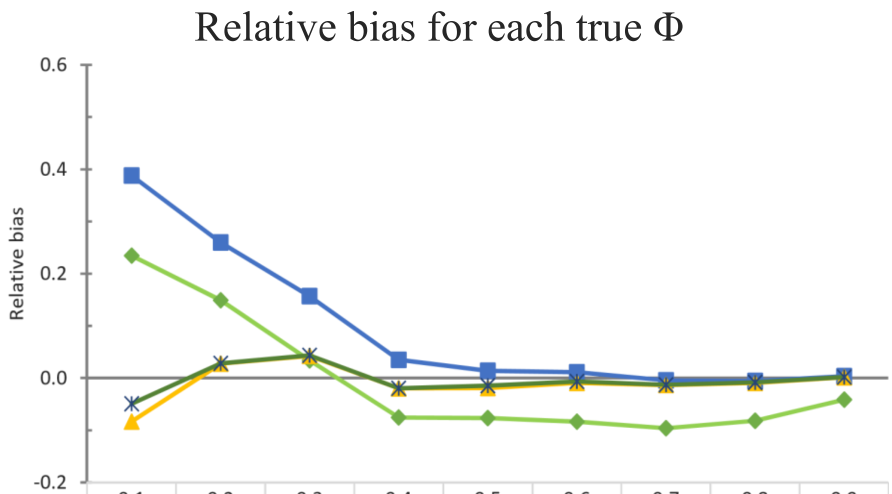

	0.1	0.2	0.3	0.4	0.5	0.6	0.7	0.8	0.9
MI1	0.235	0.149	0.034	-0.076	-0.077	-0.083	-0.096	-0.082	-0.041
MI2	0.388	0.260	0.157	0.035	0.014	0.011	-0.004	-0.006	0.004
MI3	-0.083	0.027	0.042	-0.019	-0.019	-0.010	-0.013	-0.009	0.002
MI4	-0.049	0.028	0.043	-0.020	-0.014	-0.006	-0.013	-0.009	0.002

.

Figure A2 Median absolute and relative estimation error for each model implementation over one thousand shorter univariate time series with a minimum interval length of 15 minutes (1/6th of a block). Each line represents a model implementation, and on the X-axis we have the nine different true values of φ.

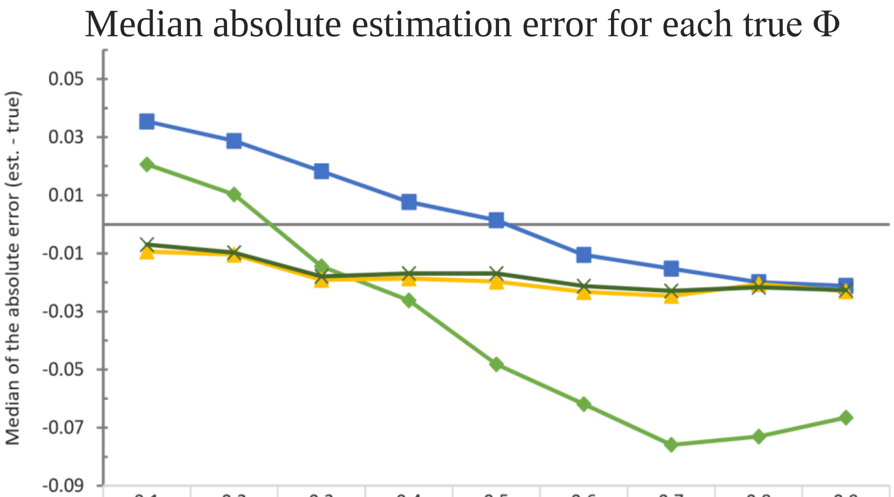

	0.1	0.2	0.3	0.4	0.5	0.6	0.7	0.8	0.9
MI1	0.021	0.010	-0.014	-0.026	-0.048	-0.062	-0.076	-0.073	-0.067
MI2	0.035	0.029	0.018	0.008	0.001	-0.010	-0.015	-0.020	-0.021
MI3	-0.009	-0.010	-0.019	-0.019	-0.020	-0.023	-0.025	-0.021	-0.023
MI4	-0.007	-0.010	-0.018	-0.017	-0.017	-0.021	-0.023	-0.022	-0.023

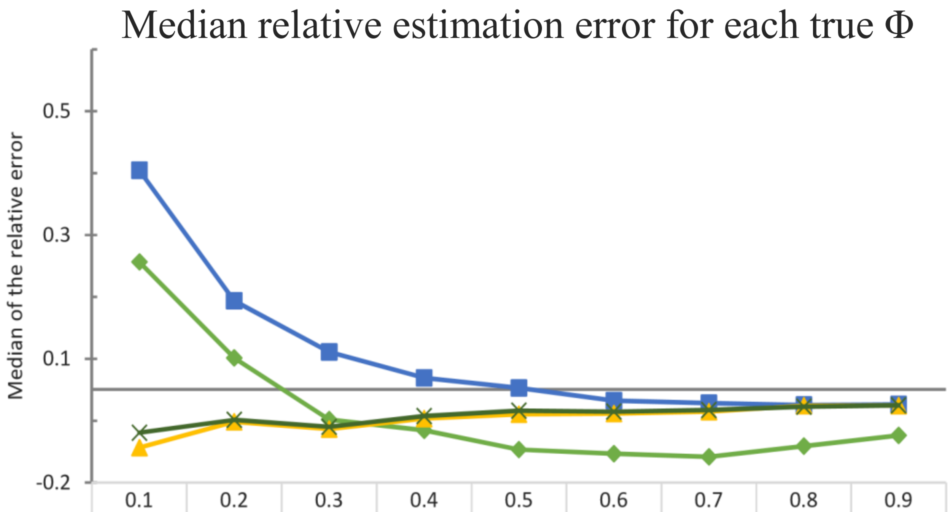

	0.1	0.2	0.3	0.4	0.5	0.6	0.7	0.8	0.9
MI1	0.207	0.052	-0.048	-0.065	-0.096	-0.103	-0.108	-0.091	-0.074
MI2	0.354	0.144	0.061	0.019	0.003	-0.017	-0.022	-0.025	-0.024
MI3	-0.094	-0.052	-0.064	-0.047	-0.039	-0.039	-0.035	-0.026	-0.026
MI4	-0.069	-0.049	-0.060	-0.042	-0.034	-0.036	-0.033	-0.027	-0.025

Figure A3 Median absolute and relative estimation error for each model implementation in the bivariate simulation with one very long time series and a minimum interval length of 15 minutes (1/6th of a block). In this graph, each stacked bar represents a model implementation and each color represents a different element of **Φ**. It can be seen that MI 1 and especially MI 2 have a greater total amount of bias (longer bars) than MI 3 and 4, especially when considering the *relative* bias. MI 2 stands out particularly because of its large positive bias in both of the autocorrelations.

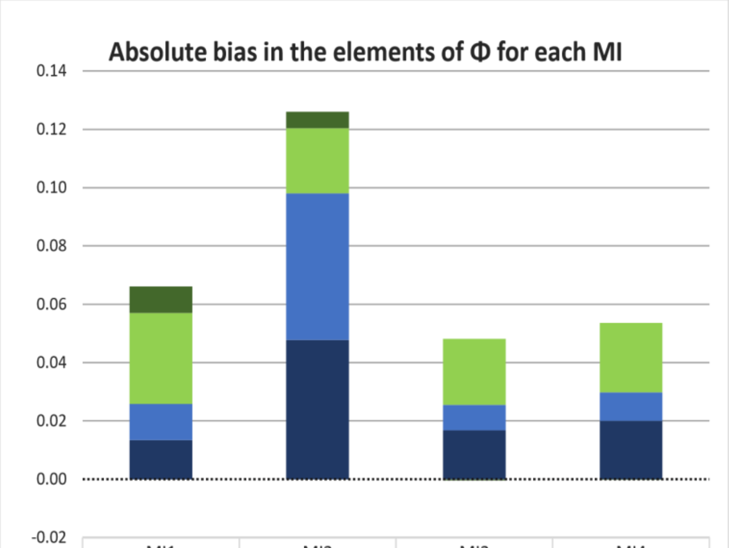

	MI1	MI2	MI3	MI4
Φ21	0.009	0.006	0.000	0.000
Φ12	0.031	0.022	0.023	0.024
Φ22	0.012	0.050	0.009	0.010
Φ11	0.013	0.048	0.017	0.020

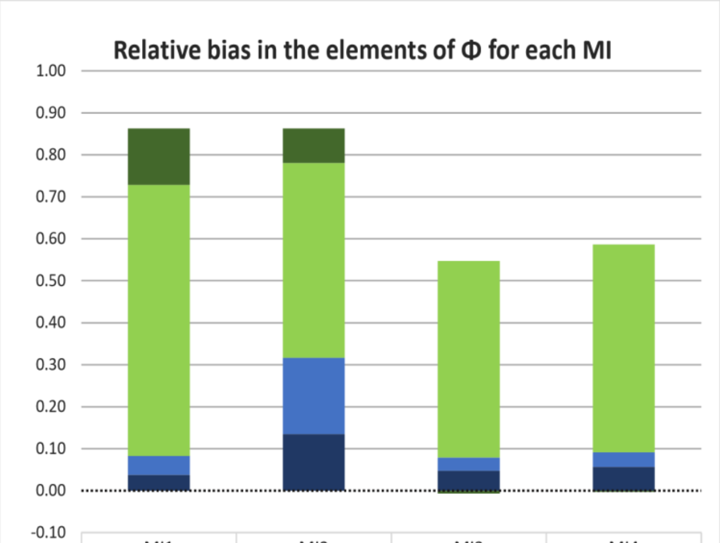

	MI1	MI2	MI3	MI4
Φ21	0.134	0.082	-0.006	-0.003
Φ12	0.646	0.464	0.468	0.495
Φ22	0.045	0.181	0.031	0.035
Φ11	0.038	0.135	0.048	0.057

.

Figure A4 Median absolute and relative estimation error for each model implementation over one thousand shorter bivariate time series with a minimum interval length of 15 minutes ($\frac{1}{6}$th of a block). Under these conditions, none of the model implementations clearly outperforms the others in terms of the total amount of (absolute or relative) estimation error in **Φ**. However, MI 2 again stands out because it is the only MI which overestimates the autocorrelations.

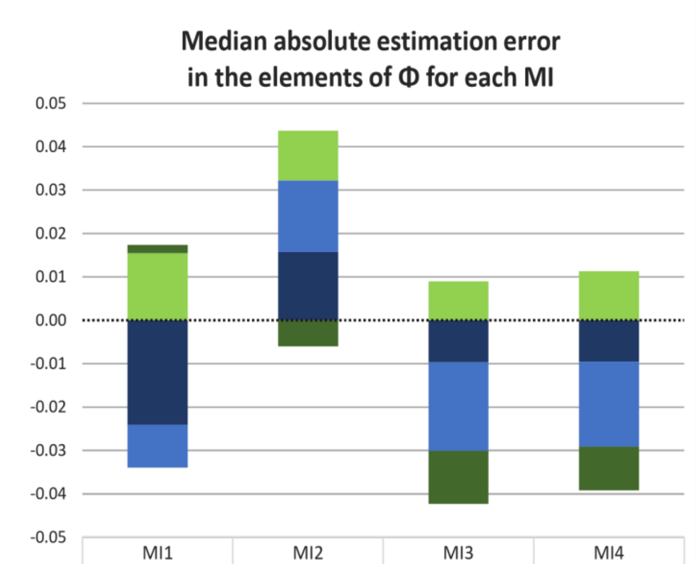

	MI1	MI2	MI3	MI4
Φ21	0.002	-0.006	-0.012	-0.010
Φ12	0.015	0.011	0.009	0.011
Φ22	-0.010	0.017	-0.020	-0.020
Φ11	-0.024	0.016	-0.010	-0.010

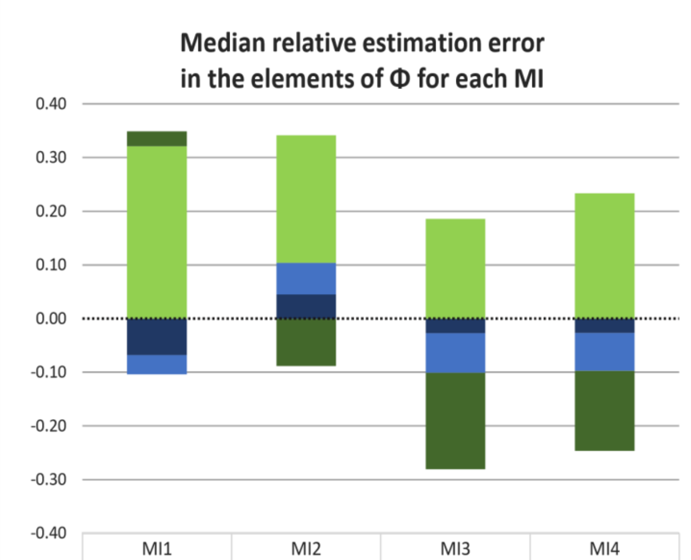

	MI1	MI2	MI3	MI4
Φ21	0.028	-0.088	-0.179	-0.149
Φ12	0.321	0.238	0.186	0.234
Φ22	-0.036	0.060	-0.074	-0.071
Φ11	-0.068	0.044	-0.027	-0.027
